# The Challenges of Managing Pediatric Diabetes and Other Endocrine Disorders During the COVID-19 Pandemic: Results From an International Cross-Sectional Electronic Survey

**DOI:** 10.3389/fendo.2021.735554

**Published:** 2021-11-05

**Authors:** Nancy Samir Elbarbary, Tiago Jeronimo dos Santos, Carine de Beaufort, Esko Wiltshire, Aman Pulungan, Andrea Enzo Scaramuzza

**Affiliations:** ^1^ Diabetes Unit, Department of Pediatrics, Faculty of Medicine, Ain Shams University, Cairo, Egypt; ^2^ Instituto Hispalense de Pediatría, Vithas Almería, Almería, Spain; ^3^ Department of Preventive Medicine and Public Health, Universidad Autónoma de Madrid, Madrid, Spain; ^4^ Diabetes & Endocrine Care Clinique Pédiatrique (DECCP), Clinique Pédiatrique/Centre Hospitalier (CH) de Luxembourg, Luxembourg, Luxembourg; ^5^ Department Pediatric Endocrinology, Free University Hospital Brussels, Brussels, Belgium; ^6^ Department of Paediatrics and Child Health, University of Otago Wellington, Wellington, New Zealand; ^7^ Department of Child Health, Capital and Coast District Health Board, Wellington, New Zealand; ^8^ Child Health Department, Faculty of Medicine Universitas Indonesia, Cipto Mangunkusumo General Hospital, Jakarta, Indonesia; ^9^ Diabetes and Endocrinology & Nutrition, Division of Pediatrics, Azienda Socio Sanitaria Territoriale (ASST) Cremona, “Ospedale Maggiore di Cremona”, Cremona, Italy

**Keywords:** COVID-19, children, diabetes, obesity and metabolic syndrome, adrenal, thyroid, growth, puberty

## Abstract

**Background:**

Frequency, dimensions, management, and outcomes of the COVID-19 pandemic in children with endocrine disorders and diabetes were assessed.

**Methods:**

A cross-sectional electronic survey was distributed to the global network of endocrine societies. Respondents’ professional and practice profiles, clinic sizes, their country of practice, and the impact of COVID-19 on endocrine diseases were investigated.

**Results:**

Respondents from 131 pediatric endocrine centers in 51 countries across all continents completed the survey. Routine check-ups and education were altered in most pediatric endocrine clinics. Over 20% of clinics experienced a shortage of critical medications or essential supplies. ICU treatment was required for patients with diabetes and COVID-19 in 21.2% of centers. In diabetes, 44% of respondents reported increased diabetic ketoacidosis episodes in newly diagnosed cases and 30% in established cases. Biopsychosocial and behavioral changes were explicitly reported to be occurring among pediatric patients with endocrine disorders.

**Conclusions:**

This large global survey conducted during the COVID-19 pandemic highlights that diabetes is more challenging to manage than any other pediatric endocrine disorder, with an increased risk of morbidity. Psychological distress due to COVID-19 needs to be recognized and addressed. The importance of close contact with healthcare professionals should be emphasized, and medical supplies should be readily available to all patients.

## Highlights

1. A previous survey found increased risk of diabetic ketoacidosis at diabetes diagnosis.
2. Over 20% of clinics faced a shortage of critical medications or supplies.
3. Patients with diabetes needed intensive care in 21.2% centers.
4. Biopsychosocial concerns were highly reported, including attempted suicide. They must be recognized and addressed.
5. During the COVID-19 pandemic, management of diabetes appears to have been more challenging than other pediatric endocrine disorders.

## 1 Introduction

The COVID-19 pandemic caused by severe acute respiratory syndrome coronavirus-2 (SARS-CoV-2) is still having a severe global impact (1). While progress has been made in immunization, there are likely to be recurrent “waves” of infection over several years until a majority of the population achieves immunity either through infection or vaccination (2–4).

While children generally have milder disease than adults (5), occasional cases of a Kawasaki-like disease linked to SARS-CoV-2 infection is now a recognized complication of COVID-19 [multisystem inflammatory syndrome (MIS) in children (MIS-C)] (6–8). However, very little is known about the impact of COVID-19 on patients with chronic conditions like endocrine diseases, especially in children (9). Most recent studies have examined endocrine disorders in adult populations (10–13). This is despite healthcare professionals (HCPs) and families requiring guidance on the clinical management of children with endocrine disorders during the challenging circumstances of the COVID-19 pandemic, especially those with suspected or confirmed COVID-19.

Here we report the outcomes of a global survey of pediatric endocrine specialist HCPs registered in the International Consortium for Pediatric Endocrinology (ICPE) database. We report the clinical characteristics of patients managed during the pandemic and highlight the knowledge and practice of HCPs working in pediatric endocrinology and the specific challenges they have faced during the pandemic. Moreover, we discuss the gaps identified between a previous survey on diabetes conducted early in the pandemic (9) and the current survey to assess and explain the frequency, dimensions, management, viewpoints, and outcomes of the COVID-19 pandemic in children, adolescents, and young people living with chronic endocrine disease.

## 2 Methods

### 2.1 Study Design and Setting

This was a cross-sectional electronic survey conducted over an eight-week period from December 3, 2020 to February 5, 2021. The survey was conducted using Google Forms (Google LLC, Mountain View, CA, United States), which allows responses to be saved and subsequently analyzed in spreadsheet format. Details of the data collection and survey methodology are described in our previous publication (9).

The target population was identified from a global network of endocrine societies, under the umbrella of the ICPE, made up of the International Society for Pediatric and Adolescent Diabetes (ISPAD), European Society for Pediatric Endocrinology (ESPE), Global Pediatric Endocrinology and Diabetes (GPED), Latin American Society of Pediatric Endocrinology (SLEP), Australasian Pediatric Endocrine Group (APEG), Asia Pacific Pediatric Endocrine Society (APPES), African Society for Pediatric and Adolescent Endocrinology (ASPAE), Arab Society for Pediatric Endocrinology Diabetes (ASPED), Chinese Society of Pediatric Endocrinology and Metabolism (CSPEM), Indian Society for Pediatric and Adolescent Endocrinology (ISPAE), Japanese Society for Pediatric Endocrinology (JSPE), Pediatric Endocrinology Society of North America (PES), and Russian Pediatric Endocrinology Group (RAE). Previous participants of the societies’ conferences, training schools, or postgraduate courses were also included.

### 2.2 The Survey

The full version of the survey is available in **Supplemental File 1**. Briefly, the survey questions were developed by six pediatric endocrinologists, and a direct web link and consent to participate in the survey was sent to ICPE members by email and social media platforms (Facebook, Twitter, and LinkedIn).

The survey questions were divided into fourteen sections that captured information on responders’ professional and practice profiles, sizes of their clinic and country of practice, and their management of the most common endocrine diseases.

The questions covered the practice and perceptions of HCPs with respect to the number of patients cared for, the organization of education sessions, the impact of the COVID-19 pandemic on daily routine, the availability of medications, frequency of acute complications, delays to diagnoses, deterioration of disease control, and the psychological impact on patients and their families. We included a few specific questions to characterize the profile of patients who tested positive for SARS-CoV-2 infection including their characteristics, clinical presentation, diagnosis, and treatment. The survey took about thirty minutes to complete.

### 2.3 Statistical Analysis

Data were analyzed using STATA 14.0 for Windows (College Station, TX, USA). The unit of analysis corresponded to a single center. Descriptive statistics were used to present demographic data and to evaluate the knowledge, attitudes, and perceptions of HCPs during the COVID-19 pandemic. Quantitative variables were described in the form of means and standard deviations (SD), and qualitative variables were described as numbers and percentages. The sum of some results exceeds the number of responses because of the option to answer as multiple choices. Some questions were open-ended and were analyzed using a coding technique, where similar answers are summarized by approximation into similar semantic content (14).

## 3 RESULTS

### 3.1 Responders’ Professional and Practice Profiles

A total of 136 responses were assessed; two responders declined to participate in the survey, and three responses were duplicates so were suppressed from analyses. A convenience sample of 131 pediatric endocrine centers from 51 countries across all continents participated in the study and were included in the final analysis. The country of origin of each responder and their professional background, center setting, and size are shown in **Table 1**.

**Table 1:** Endocrine clinical center characteristics and staff profiles.

Characteristics (n respondents)	Respondents (%)
**Centers by country (131)**	
United Sates of America	11 (8.4)
Spain	9 (6.9)
Philippines	8 (6.1)
Germany	7 (5.3)
Egypt, Italy	6 (4.6) each
Argentina, Brazil, United Kingdom	5 (3.8) each
Canada, Greece	4 (3.0) each
India, Japan, Netherlands, Portugal	3 (2.3) each
Belgium, Bulgaria, Congo, Denmark, Indonesia, Iran, Malaysia, Mexico, New Zealand, Peru, Serbia and Montenegro, Sweden, Turkey	2 (1.5) each
Australia, Austria, Bangladesh, Chile, Cyprus, Finland, Georgia, Hong Kong, Hungary, Iceland, Iraq, Ireland, Israel, Lebanon, Luxembourg, Malta, Netherlands Antilles, Poland, Romania, Slovenia, Sudan Taiwan, Ukraine	1 (0.8) each
**Current clinical role (131)**	
Pediatric endocrinologist/diabetologist	109 (83.2)
Pediatrician with interest in endocrinology	15 (11.4)
Resident or fellow or trainee in pediatrics/pediatric endocrinology or diabetology or diabetes researcher	4 (3.0)
Adult physician looking after pediatric or adolescent patients	2 (1.5)
Nurse practitioner/registered nurse	1 (0.8)
**Clinical setting (131)**	
University/academic hospital or clinic	64 (48.9)
Public/governmental hospital or clinic	41 (31.3)
Private hospital or clinic	25 (19.1)
Primary care center	1 (0.8)
**Estimate case-mix, patients by endocrine disorders**	
Type 1 diabetes	
<100	46 (40.7%)
100-250	39 (34.5%)
251-500	16 (14.2%)
>500	12 (10.6%)
Type 2 diabetes	
≤50	45 (93.7%)
51-100	1 (2.1%)
>100	3 (6.2%)
Other forms of diabetes	
≤50	41 (91.1%)
51-100	1 (2.2%)
>100	3 (6.7%)
Obesity and metabolic syndrome	
≤50	37 (58.7%)
51-100	8 (12.7%)
>100	18 (28.6%)
Hyperinsulinemic hypoglycemia	
≤50	39 (97.5%)
>50	1 (2.5%)
Thyroid	
≤50	49 (55.1%)
51-100	16 (18.0%)
>100	24 (26.9%)
Adrenal	
≤50	51 (79.7%)
50-100	10 (15.6%)
>100	3 (7.8%)
Bone metabolism	
≤50	26 (83.9%)
>50	5 (16.1%)
Pituitary and other CNS disorder	
≤50	55 (90.2%)
>50	6 (9.8%)
Growth	
≤50	44 (61%)
51-100	515 (21%)
>100	13 (18%)
Pubertal	
≤50	50 (73.5%)
51-100	12 (17.6%)
>100	6 (8.8%)
Others: Gender dysphoria	
≤50	1 (50%)
>50	1 (50%)

### 3.2 Overall Outcomes From Pediatric Endocrine Disorders

During the COVID-19 pandemic, routine follow-up visits and education were altered in most pediatric endocrine centers, with care and disease literacy most often delivered face-to-face (F2F) using appropriate personal protective equipment (PPE) and to a lesser extent using telephone and video consultations. For diabetes care, F2F care was restricted to only one caregiver, while for hyperinsulinemic hypoglycemia (HH) and bone metabolism disorders, over one-fifth of centers maintained care as usual. Unsurprisingly, in most centers, contact with the diabetes or endocrine team was limited due to the fear of COVID-19 by the families themselves.

Over 20% of responder centers reported shortages of several supplies specific for diabetes, HH, adrenal, and bone metabolism disorders: glucose test stripes (14.1%), blood glucose sensors (12.4%), basal (11.5%) and bolus (10.6%) insulin, diazoxide (19.5%), hydrocortisone (25.7%), and calcitriol (9.4%). In addition, genetic testing and imaging were in part affected in centers managing monogenic diabetes (14.3%) and bone metabolism disorders (9.4%) (**Tables 2**, **3**).

**Table 2:** Assessment of pediatric diabetes care during the COVID-19 pandemic by clinical centers.

	Type 1 diabetes (n=113)	Type 2 diabetes (n=52)	Other forms of diabetes (n=47)
**Estimate proportion of delayed diagnose due to COVID-19**	45.3%	17.3%	17.0%
**Estimate perception of worsening disease management**	31.0%	67.3%	29.8%
**Use of technologies among patients, %**			
• Insulin pump		N/A	N/A
○ Less than 10	39 (34.5%)		
○ 10-25	17 (15.0%)		
○ 26-50	23 (20.3%)		
○ 51-75	23 (20.3%)		
○ 76-100	11 (9.7%)		
• CGMS		N/A	N/A
○ Less than 10	38 (33.6%)		
○ 10-25	28 (24.8%)		
○ 26-50	21 (18.6%		
○ 51-75	21 (18.6%)		
○ 76-100	5 (4.4%)		
• Flash GMS		N/A	N/A
○ Less than 10	35 (31.0%)		
○ 10-25	31 (27.4%)		
○ 26-50	18 (15.9%)		
○ 51-75	19 (16.8%)		
○ 76-100	10 (8.8%)		
**Testing**			
• COVID-19 tests for newly diagnosed.	71 (62.8%)	N/A	N/A
• Positivity			
○ No positives with standardized tests	56 (49.6%)	N/A	N/A
○ Less than 25%	54 (47.8%)		
○ 26-50%	1 (0.9%)		
○ More than 75%	2 (1.8%)		
• COVID-19 tests in DKA cases	79 (69.9%)	N/A	N/A
• Positivity with standardized tests			
○ Less than 10%			
**Testing**			
• COVID-19 tests for newly diagnosed.	71 (62.8%)	N/A	N/A
• Positivity			
○ No positives with standardized tests	56 (49.6%)	N/A	N/A
○ Less than 25%	54 (47.8%)		
○ 26-50%	1 (0.9%)		
○ More than 75%	2 (1.8%)		
• COVID-19 tests in DKA cases	79 (69.9%)	N/A	N/A
• Positivity with standardized tests			
○ Less than 10%	75 (87.2%)	N/A	N/A
○ 10-25%	6 (7.0%)		
○ 26-50%	2 (2.3%)		
○ More than 75%	3 (3.5%)		
**Diabetic ketoacidosis episodes**			
• Increase of newly-onset cases	50 (44.2%)	N/A	N/A
• Increase in stablished cases	34 (30.1%)	N/A	N/A
• Proportion of DKA episodes			
○ 0-25%	66 (58.4%)	N/A	N/A
○ 26-50%	16 (14.1%)		
○ 51-75%	20 (17.7%)		
○ 76-100%	11 (9.7%)		
• Proportion of mild DKA		N/A	N/A
○ 0-25%	64 (56.6%)		
○ 26-50%	31 (27.4%)		
○ 51-75%	14 (2.4%)		
○ 76-100%	4 (3.6%)		
• Proportion of moderate DKA		N/A	N/A
○ 0-25%	68 (60.2%)		
○ 26-50%	33 (29.2%)		
○ 51-75%	9 (8.0%)		
○ 76-100%	3 (2.8%)		
• Proportion of severe DKA		N/A	N/A
○ 0-25%	81 (71.7%)		
○ 26-50%	15 (13.3%)		
○ 51-75%	15 (13.3%)		
○ 76-100%	2 (1.8%)		
• Perception of worsening episodes	48 (42.5%)	N/A	N/A
**Severe Hypoglycemia episodes**			
• Increase of SH episodes	7 (6.2%)	N/A	N/A
**Routine check-up**			
• As usual, no changes	18 (15.9%)	9 (17.3%)	9 (19.2%)
• Sent SMS and emails for consultation.	34 (30%)	10 (19.2%)	13 (27.6%)
• Apps	19 (16.8%)	6 (11.3%)	12 (25.5%)
• Telephone consultations	73 (64.6%)	27 (51.9%)	27 (57.4%)
• Video consultations	50 (44.2%)	23 (44.2%)	18 (38.3%)
• Face to face consultation with appropriate personal protective equipment restricted to just one parent/caregiver	75 (66.3%)	N/A	N/A
• Face to face consultation with appropriate personal protective equipment where all caregivers are allowed to attend	15 (13.2%)	34 (65.4%)	28 (59.5%)
• No consultation during complete lockdown or postponing it to annual visits	2 (1.7%)	1 (1.9%)	0
• Limited contact with diabetes team because of COVID-19 fear	85 (75.2%)	39 (75.0%)	34 (72.3%)
**Daily routine**			
• Maintenance of physical activity			
○ Less than 10%	23 (20.3%)	N/A	N/A
○ 10-25%	47 (41.6%		
○ 26-50%	21 (18.6%)		
○ 51-75%	12 (10.6%)		
○ 76-100%	10 (8.8%)		
• Worsening of dietary choices			
○ Less than 10%	26 (23.0%)		N/A
○ 10-25%	31 (27.4%)		
○ 26-50%	37 (32.7%)		
○ 51-75%	8 (7.1%)		
○ 76-100%	11 (9.7%)	N/A	
• Increase of body weight			
○ Less than 10%	23 (20.3%)		N/A
○ 10-25%	28 (24.8%)		
○ 26-50%	36 (31.9%)		
○ 51-75%	18 (15.9%)		
○ 76-100%	8 (7.1%)		
• Extra dose of insulin			
○ Yes			
○ No	N/A		
○ Not on insulin therapy		N/A	N/A
• Average glycemic control during pandemic			
○ Mostly improved	21 (18.6%)		
○ Mostly maintained same level	57 (50.4%)		
○ Mostly worsened	35 (31.0%)		N/A
• Regular use of anti-hypertensive			
○ ACE inhibitor	N/A		
○ B-blocker			
○ Ca channel blocker		18 (34.6%)	N/A
○ Control with salt-free diet		18 (34.6%)	
○ None		16 (30.8%)	
		N/A	
		26 (76.5%)	
		1 (2.9%)	
		2 (5.9%)	
		1 (2.9%)	
		5 (14.7%)	
**School activities**			
• Parental concerns to return to school activities	90 (79.6%)	N/A	N/A
• Specific school guidelines during pandemic	90 (79.6%)		
**Patient and family education**			
• As usual, no changes	8 (7%)	6 (11.5%)	3 (6.4%)
• By telephone	52 (46%)	22 (42.3%)	30 (63.8%)
• Video consultations	49 (43%)	20 (38.5%)	18 (38.3%)
• Apps/digital platforms	21 (19%)	14 (26.9%)	14 (29.8%)
• Face to face education wearing appropriate personal protective equipment	97 (86%)	41 (78.8%)	39 (83.0%)
**Supplies**			
• Refill prescription		N/A	N/A
○ Refill prescription every month	12 (10.6%)		
○ Refill prescription every 3 months or less	53 (46.7%)		
○ Refill prescription every 6 months or less	14 (12.4%)		
○ Refill prescription every year or less	9 (8.0%)		
○ As required by patient	13 (11.5%)		
○ Automatic refill prescription from pharmacy	5 (4.4%)		
○ I am not directly involved with prescription	7 (6.2%)		
• Shortage of supplies			
○ Yes	25 (22.1%)	7 (13.5%)	6 (12.8%)
○ No, everything was secured	78 (69.0%)	33 (63.5%)	35 (74.5%)
○ I am not aware of situation	10 (8.9%)	12 (23.1%)	6 (12.8%)
• Item under shortage			
○ Basal insulin	13 (11.5%)	2 (12.5%)	1 (7.1%)
○ Bolus insulin	12 (10.6%)	0	0
○ Glucose test strips	16 (14.1%)	3 (18.7%)	4 (28.6%)
○ Blood glucose sensors	14 (12.4%)	0	3 (21.4%)
○ Insulin pump supplies	6 (5.3%)	0	0
○ Ketone test strips	8 (7.1)	0	0
○ Oral medication	0	1 (6.2%)	1 (7.1%)
○ Genetic tests	0	1 (6.2%)	2 (14.3%)
**Characteristics of COVID-19 cases**			
• Estimate mean age, years	11.4 (SD 3.6) (n=60)	13.6 (SD 2.3) (n=9)	N/A
• Estimate % boys	47.3 (SD 24.2) (n=46)	53.4 (SD 24.9) (n=11)	
• Estimate % girls	52.2 (SD 24.3) (n=46)	46.6 (SD 25.0) (n=11)	
• Estimate mean duration of disease, years	3.5 (SD 2.3) (n=55)	1.2 (SD 1.4) (n=13)	
• Estimate mean HbA1c value, %	8.5 (1.6) (n=50)	7.7 (SD 3.2) (n=10)	
• BMI, Kg/m²	N/A	30.2 (SD 3.0) (n=8)	
• **Presence of comorbidities**			
○ Asthma	32 (28.3%)	15 (28.8%)	5 (10.6%)
○ Cancer	3 (2.6%)	2 (3.8%)	4 (8.5%)
○ Obesity	44 (38.9%)	48 (92.3%)	10 (21.3%)
○ Hypertension	19 (16.8%)	23 (44.2%)	1 (2.1%)
○ Heart disease	3 (2.6%)	0	2 (4.2%)
○ Kidney disease	15 (13.3%)	3 (5.8%)	8 (17.0%)
○ Neurological disease	2 (1.7%)	0	2 (4.2%)
○ Celiac disease	10 (8.8%)	0	0
○ Hypothyroidism	8 (7.1%)	0	0
○ Cystic fibrosis or bronchial dysplasia	11 (9.7%)	2 (3.8%)	12 (25.5%)
○ Other: Allergy, other autoimmune disease, dyslipidemia, Polycystic ovary syndrome, Lupus, Anemia	4 (3.5%)	2 (3.8%)	2 (4.2%)
○ No	32 (28.3%)	4 (7.7%)	18 (38.3%)
**Psychological concerns**			
• Anxiety	69 (61.1%)	31 (59.6%)	24 (51.1%)
• Parenting stress	61 (54.0%)	22 (42.3%)	17 (36.2%)
• Depression	42 (37.2%)	24 (46.1%)	14 (29.8%)
• Insomnia/hypersomnia or other sleep disruption	36 (31.8%)	13 (25.0%)	5 (10.6%)
• Eating disorder	25 (22.1%)	15 (28.8%)	5 (10.6%)
• Panic attacks	17 (15.0%)	11 (21.1%)	7 (14.9%)
• Suicide attempt	6 (5.3%)	2 (3.8%)	1 (2.1%)
• Patient or caregivers have improved the mood	4 (3.5%)	0	1 (2.1%)
• None have had psychological problems so far	31 (27.4%)	15 (28.8%)	20 (42.5%)

N/A, not applicable.

**Table 3:** Assessment of pediatric endocrine care other than diabetes during COVID-19 pandemic by clinical centers.

	Obesity and Metabolic Syndrome (n=65)	Hyperinsulinemic hypoglycemia (n=41)	Thyroid disorders (n=91)	Adrenal disorders (n=66)	Bone metabolism disorders (n=32)	Pituitary and other CNS disorders (n=62)	Growth disorders (n=73)	Pubertal disorders (n=69)
**Estimated proportion of delayed diagnoses due to COVID-19**	41.5%	9.8%	22.0%	15.1%	18.7%	21.0%	39.4%	37.7%
**Estimated proportion of increase in severity**	N/A	7.3%	N/A	4.6%	N/A	N/A	N/A	N/A
**Estimate perception of worsening disease management**	83.1%	24.4%	22.0%	36.4%	31.2%	27.4%	35.6%	34.8%
**Routine check-up**								
• As usual, no changes	11 (16.9%)	12 (29.3%)	17 (18.7%)	9 (13.6%)	7 (21.9%)	11 (17.7%)	10 (13.7%)	13 (18.8%)
• Sent SMS and emails for consultation	15 (23.1%)	10 (24.4%)	24 (26.4%)	18 (27.3%)	10 (31.2%)	17 (27.4%)	22 (30.1%)	18 (26.1%)
• Apps	5 (7.7%)	7 (17.1%)	7 (7.7%)	7 (10.1%)	7 (21.9%)	5 (8.1%)	8 (10.9%)	8 (11.6%)
• Telephone consultations	36 (55.4%)	20 (48.8%)	53 (58.2%)	39 (59.1%)	16 (50.0%)	37 (59.7%)	44 (60.3%)	36 (52.2%)
• Video consultations	19 (29.2%)	9 (21.9%)	27 (29.7%)	21 (31.8%)	10 (31.2%)	20 (32.3%)	23 (31.5%)	22 (31.9%)
• Face to face consultation with appropriate personal protective equipment	43 (66.1%)	23 (56.1%)	60 (65.9%)	51 (77.3%)	19 (59.4%)	41 (66.1%)	53 (72.6%)	51 (73.9%)
• No consultation during complete lockdown or postponing it to annual visits	1 (1.5%)	0	1 (1.1%)	1 (1.5%)	0	1 (1.6%)	1 (1.4%)	0
• Limited contact with endocrine team because of COVID-19 fear	59 (90.7%)	26 (63.4%)	67 (73.6%)	45 (68.2%)	22 (68.7%)	39 (62.9%)	49 (67.1%)	48 (69.6%)
**Patient and family education**								
• As usual, no changes	3 (4.6%)	3 (7.3%)	7 (7.7%)	3 (4.5%)	2 (6.1%)	2 (3.2%)	5 (6.8%)	4 (5.6%)
• By telephone	34 (52.3%)	25 (61.0%)	53 (58.2%)	36 (54.5%)	20 (62.5%)	35 (56.4%)	39 (53.4%)	34 (49.3%)
• Video consultations	21 (32.3%)	11 (26.8%)	28 (30.8%)	25 (37.9%)	11 (34.4%)	22 (35.5%)	25 (34.2%)	23 (33.3%)
• Apps/digital platforms	10 (15.4%)	8 (19.5%)	12 (13.2%)	11 (16.7%)	8 (25.0%)	9 (14.5%)	12 (16.4%)	11 (15.9%)
• Face to face education wearing appropriate personal protective equipment	47 (72.3%)	33 (80.5%)	68 (74.2%)	54 (81.8%)	26 (81.2%)	50 (80.7%)	59 (80.8%)	56 (81.2%)
**Supplies**								
• Shortage of supplies								
○ Yes	4 (6.1%)	9 (21.9%)	9 (9.9%)	16 (24.2%)	7 (21.9%)	11 (17.7%)	12 (16.4%)	12 (17.4%)
○ No, everything was secured	48 (73.9%)	26 (63.4%)	72 (79.1%)	41 (62.1%)	20 (62.5%)	40 (64.5%)	53 (72.6%)	48 (69.6%)
○ I am not aware of situation	13 (20.0%)	6 (14.6%)	10 (11.0%)	9 (13.6%)	5 (15.7%)	11 (17.7%)	8 (11.0%)	9 (13.0%)
• Item under shortage								
○ Oral/nasal medications (e.g., metformin, diazoxide, levothyroxine, methimazole, hydrocortisone, fludrocortisone, calcitriol, desmopressin, estrogen)	2 (3.1%)	8 (19.5%)	9 (9.9%)	17 (25.7%)	3 (9.4%)	8 (12.9%)	0	1 (1.4%)
○ Injectable medications (e.g., insulin, octreotide, glucagon, bisphosphonate, GnRHa, rhGHa)	1 (1.5%)	2 (4.9%)	0	0	2 (6.2%)	2 (3.2%)	10 (13.7%)	11 (15.9%)
○ Topic medications (e.g., estrogen)	0	0	0	0	0	1 (1.6%)	0	0
○ Test strips	1 (1.5%)	1 (2.4%)	N/A	N/A	N/A	N/A	N/A	N/A
○ Syringe	0	1 (2.4%)	0	0	0	0	1 (1.4%)	0
○ Genetic testing /imaging	0	1 (2.4%)	1 (1.1%)	1 (1.5%)	3 (9.4%)	2 (3.2%)	3 (4.1%)	2 (2.9%)
• **Presence of comorbidities**								
○ Asthma	27 (41.5%)	2 (4.9%)	15 (16.5%)	6 (9.1%)	1 (3.1%)	4 (6.4%)	16 (21.9%)	10 (14.5%)
○ Cancer	2 (3.1%)	0	5 (5.5%)	2 (3.0%)	1 (3.1%)	11 (17.7%)	4 (5.5%)	5 (7.2%)
○ Obesity	N/A	6 (14.6%)	27 (29.7%)	11 (16.7%)	9 (28.1%)	29 (46.8%)	21 (28.8%)	25 (36.2%)
○ Hypertension	42 (64.6%)	0	4 (4.4%)	5 (7.5%)	0	2 (3.2%)	3 (4.1%)	1 (1.4%)
○ Heart disease	4 (6.1%)	0	5 (5.5%)	2 (3.0%)	0	3 (4.8%)	9 (12.3%)	3 (4.3%)
○ Kidney disease	5 (7.7%)	3 (7.3%)	2 (2.2%)	1 (1.5%)	10 (31.2%)	3 (4.8%)	10 (13.7%)	6 (8.7%)
○ Neurological disease	0	2 (4.9%)	0	0	1 (3.1%)	0	0	2 (2.9%)
○ Celiac disease	0	0	6 (6.5%)	0	0	0	0	1 (1.4%)
○ Hypothyroidism	2 (3.1%)	0	N/A	1 (1.5%)	0	1 (1.6%)	0	1 (1.4%)
○ Cystic fibrosis or bronchial dysplasia	0	0	0	0	1 (3.1%)	1 (1.6%)	7 (9.6%)	0
○ Other: allergy, dyslipidemia, polycystic ovary syndrome, lupus, anemia, obstructive sleep apnea, hepatic steatosis, bowel complication, other autoimmune or genetic disease	7 (10.8)	1 (2.4%)	7 (7.7%)	10 (15.1%)	1 (3.1%)	2 (3.2%)	5 (6.8%)	3 (4.3%)
○ No	10 (15.4%)	28 (68.3%)	44 (48.3)	36 (54.5%)	11 (34.4%)	26 (41.9%)	31 (42.5%)	30 (43.5%)
**Use of medications**								
• **Anti-hypertensive**		N/A	N/A	N/A	N/A	N/A	N/A	N/A
○ ACE inhibitors I/II	32 (49.2%)							
○ Beta-blocker	2 (3.1%)							
○ Ca channel blocker	5 (7.7%)							
○ Salt-free diet	2 (3.1%)							
○ No treatment	25 (38.5%)							
○ Continuation of anti-hypertensive	40 (61.5%)							
○ No complication with anti-hypertensive use	40 (61.5%)							
○ Maintenance of treatment during COVID19	40 (61.5%)	N/A	N/A	N/A	N/A	N/A	N/A	N/A
• Management of adrenal crisis	N/A	N/A	N/A		N/A	N/A	N/A	N/A
○ Fluid and electrolyte resuscitation				1 (1.5%)				
○ Ample doses of glucocorticoids				48 (72.7%)				
○ Chronic glucocorticoid and mineralocorticoid replacement				9 (13.6%)				
○ Treatment of the precipitating illness				1 (1.5%)				
**Psychological concerns**								
• Anxiety	41 (63.1%)	18 (43.9%)	33 (36.3%)	23 (34.8%)	12 (37.5%)	27 (43.5%)	27 (37.0%)	30 (43.5%)
• Parenting stress	35 (53.8%)	15 (36.6%)	28 (30.8%)	24 (36.4%)	12 (37.5%)	18 (29.0%)	23 (31.5%)	25 (36.2%)
• Depression	32 (49.2%)	6 (14.6%)	17 (18.7%)	12 (18.2%)	7 (21.9%)	12 (19.3%)	11 (15.1%)	8 (11.6%)
• Insomnia/hypersomnia or other sleep disruption	22 (33.8%)	5 (12.2%)	14 (15.4%)	6 (9.1%)	5 (15.6%)	9 (14.5%)	13 (17.8%)	11 (15.9%)
• Eating disorder	32 (49.2%)	4 (9.7%)	17 (18.7%)	5 (7.5%)	3 (9.4%)	10 (16.1%)	14 (19.2%)	12 (17.4%)
• Panic attacks	7 (10.8%)	5 (12.2%)	8 (8.8%)	4 (6.1%)	3 (9.4%)	7 (11.3%)	4 (5.5%)	8 (11.6%)
• Suicide attempt	3 (4.6%)	1 (2.4%)	1 (1.1%)	2 (3.0%)	1 (3.1%)	1 (1.6%)	1 (1.4%)	0
• Patient or caregivers have improved the mood	0	0	1 (1.1%)	1 (1.5%)	0	0	1 (1.4%)	0
• None have had psychological problems so far	15 (23.1%)	22 (53.6%)	41 (45.5%)	33 (50.0%)	14 (43.7%)	31 (50.0%)	36 (49.3%)	32 (46.4%)

N/A, not applicable.

The percentages of centers that had patients affected by COVID-19 are shown in **Table 2** for all forms of diabetes and in **Table 3** for other endocrine disorders. Patients with diabetes were most affected by COVID-19, with more severe symptoms, probably due to a higher percentage of comorbidities (**Table 2**) than patients with other endocrine diseases (**Table 3**). Of note, patients with bone metabolism disorders also seemed to be more susceptible to COVID-19 infection due to concurrent kidney disease (31.2%).

**Figure 1:**
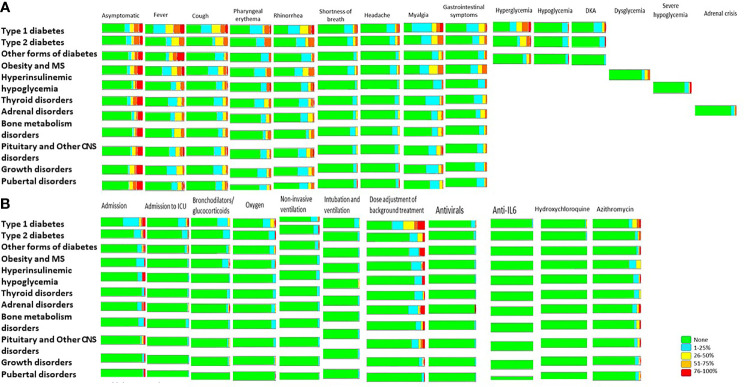
Proportion of general and specific symptomatology **(A)** and requirement for specific therapeutic management **(B)** in COVID-19 patients attending pediatric endocrine centers. MS, metabolic syndrome; CNS, central nervous system; DKA, Diabetic ketoacidosis; ICU, Intensive Care Unit; IL-6, Interleukin-6.

Among patients with COVID-19, most were asymptomatic or had mild to moderate symptoms (**Tables 4**, **5**). However, in centers where patients with type 1 diabetes had a positive COVID-19 test, symptoms appeared to be more frequent and severe than in centers with patients with other endocrine conditions (**Figure 1A**, **Tables 4**, **5**). No deaths were reported for any endocrine condition.

**Table 4:** Symptoms, complications, and outcomes of pediatric diabetes cases during the COVID-19 pandemic by clinical center.

	Type 1 diabetes (n=113)	Type 2 diabetes (n=52)	Other forms of diabetes (n=47)
**Symptoms and/or complications among COVID-19 cases**			
• Asymptomatic			
○ None	63 (55.7%)	31 (59.6%)	30 (63.8%)
○ 1-25%	20 (17.7%)	6 (11.5%)	3 (6.4%)
○ 26-50%	10 (8.8%)	3 (5.8%)	4 (8.5%)
○ 51-75%	11 (9.7%)	8 (15.4%)	4 (8.5%)
○ 76-100%	9 (8.0%)	4 (7.7%)	6 (12.8%)
• Fever			
○ None	44 (38.9%)	33 (63.5%)	33 (70.2%)
○ 1-25%	26 (23%)	5 (9.6%)	7 (14.9%)
○ 26-50%	17 (15.1%)	8 (15.4%)	4 (8.5%)
○ 51-75%	14 (12.4%)	6 (11.5%)	3 (6.4%)
○ 76-100%	12 (10.6%)	0	0
• Cough			
○ None	43 (38.05%)	34 (65.4%)	33 (70.2%)
○ 1-25%	28 (24.8%)	3 (5.8%)	9 (19.1%)
○ 26-50%	24 (21.2%)	10 (19.2%)	3 (6.4%)
○ 51-75%	11 (9.7%)	5 (9.6%)	2 (4.3%)
○ 76-100%	7 (6.2%)	0	0
• Pharyngeal erythema			
○ None	66 (58.4%)	37 (71.1%)	38 (80.8%)
○ 1-25%	26 (23%)	8 (15.4%)	7 (14.9%)
○ 26-50%	5 (4.5%)	0	0
○ 51-75%	12 (10.6%)	7 (13.5%)	2 (4.3%)
○ 76-100%	4 (3.5%)	0	0
• Rhinorrhea			
○ None	56 (49.6%)	34 (65.4%)	35 (74.5%)
○ 1-25%	23 (20.3%)	9 (17.3%)	9 (19.1%)
○ 26-50%	21 (18.6%)	3 (5.8%)	1 (2.1%)
○ 51-75%	7 (6.2%)	6 (11.5%)	2 (4.3%)
○ 76-100%	6 (5.3%)	0	0
• Shortness of breath			
○ None	75 (66.4%)	39 (75.0%)	42 (89.4%)
○ 1-25%	27 (23.9%)	7 (13.5%)	4 (8.5%)
○ 26-50%	6 (5.3%)	3 (5.8%)	1 (2.1%)
○ 51-75%	3 (2.6%)	3 (5.8%)	0
○ 76-100%	2 (1.8%)	0	0
• Headache			
○ None	50 (44.2%)	35 (67.3%)	39 (83.0%)
○ 1-25%	35 (31.0%)	8 (15.4%)	4 (8.5%)
○ 26-50%	13 (11.5%)	3 (5.8%)	2 (4.3%)
○ 51-75%	9 (8.0%)	6 (11.6%)	2 (4.3%)
○ 76-100%	6 (5.3%)	0	0
• Myalgia			
○ None	52 (46.0%)	33 (63.5%)	38 (80.8%)
○ 1-25%	34 (30.1%)	9 (17.3%)	5 (10.6%)
○ 26-50%	13 (11.5%)	5 (9.6%)	3 (6.4%)
○ 51-75%	7 (6.2%)	5 (9.6%)	1 (2.1%)
○ 76-100%	7 (6.2%)	0	0
• Gastrointestinal pain			
○ None	56 (49.6%)	34 (65.4%)	37 (78.7%)
o1-25%	33 (29.2%)	9 (17.3%)	7 (14.9%)
○ 26-50%	17 (15.0%)	6 (11.5%)	1 (2.1%)
○ 51-75%	5 (4.4%)	3 (5.8%)	2 (4.3%)
○ 76-100%	2 (1.8%)	0	0
• Hyperglycemia			
○ None	59 (52.2%)	36 (69.3%)	38 (80.8%)
○ 1-25%	17 (15.0%)	3 (5.8%)	4 (8.5%)
○ 26-50%	15 (13.3%)	8 (15.4%)	3 (6.4%)
○ 51-75%	15 (13.3%)	4 (7.7%)	2 (4.3%)
○ 76-100%	7 (6.2%)	1 (1.9%)	0
• Hypoglycemia			
○ None	95 (84.1%)	45 (86.5%)	43 (91.5%)
○ 1-25%	15 (13.3%)	6 (11.5%)	3 (6.4%)
○ 26-50%	0	1 (1.9%)	0
○ 51-75%	3 (2.6%)	0	0
○ 76-100%	0	0	1 (2,1%)
• Diabetic ketoacidosis			
○ None	80 (70.8%)	44 (84.7%)	45 (95.6%)
○ 1-25%	22 (19.5%)	7 (13.5%)	2 (4.4%)
○ 26-50%	6 (5.3%)	0	0
○ 51-75%	4 (3.5%)	0	0
○ 76-100%	1 (0.9%)	1 (1.9%)	0
**Outcomes for COVID-19 cases**			
• Admission			
○ None	67 (59.3%)	41 (78.8%)	40 (85.1%)
○ 1-25%	32 (28.3%)	7 (13.5%)	4 (8.5%)
○ 26-50%	5 (4.4%)	1 (1.9%)	1 (2.1%)
○ 51-75%	2 (1.8%)	1 (1.9%)	1 (2.1%)
○ 76-100%	7 (6.2%)	2 (3.8%)	1 (2.1%)
• Admission to intensive care unit			
○ None	89 (78.8%)	45 (86.5%)	43 (91.5%)
○ 1-25%	18 (15.9%)	6 (11.5%)	2 (4.3%)
○ 26-50%	2 (1.8%)	0	1 (2.1%)
○ 51-75%	2 (1.8%)	1 (1.9%)	1 (2.1%)
○ 76-100%	2 (1.8%)	0	0
• Need for bronchodilators and glucocorticoids			
○ None	85 (75.2%)	45 (86.5%)	44 (93.6%)
○ 1-25%	23 (20.4%)	7 (13.5%)	2 (4.3%)
○ 26-50%	5 (4.4%)	0	1 (2.1%)
○ 51-75%	0	0	0
○ 76-100%	0	0	0
• Need for oxygen			
○ None	81 (71.7%)	44 (84.6%)	40 (85.1%)
○ 1-25%	19 (16.8%)	7 (13.5%)	5 (10.6%)
○ 26-50%	9 (8.0%)	0	1 (2.1%)
○ 51-75%	1 (0.9%)	0	1 (2.1%)
○ 76-100%	3 (2.7%)	1 (1.9%)	0
• Need for non-invasive ventilation			
○ None	95 (84.1%)	46 (88.5%)	43 (91.5%)
○ 1-25%	14 (12.4%)	5 (9.6%)	3 (6.4%)
○ 26-50%	3 (2.7%)	0	0
○ 51-75%	1 (0.9%)	0	1 (2.1%)
○ 76-100%	0	1 (1.9%)	0
• Need for intubation and ventilation			
○ None	102 (90.3%)	50 (96.1%)	46 (97.9%)
○ 1-25%	10 (8.9%)	2 (3.8%)	1 (2.1%)
○ 26-50%	1 (0.9%)	0	0
○ 51-75%	0	0	0
○ 76-100%	0	0	0
• No need for specific treatments			
○ None	60 (53.1%)	33 (63.5%)	36 (76.6%)
○ 1-25%	10 (8.9%)	3 (5.8%)	3 (6.4%)
○ 26-50%	8 (7.1%)	2 (3.8%)	0
○ 51-75%	13 (11.5%)	5 (9.6%)	4 (8.5%)
○ 76-100%	22 (19.5%)	9 (17.3%)	4 (8.5%)
• Increased insulin dosage/other treatment adjustment			
○ None	49 (43.4%)	38 (73.1%)	34 (72.4%)
○ 1-25%	20 (17.7%)	7 (13.5%)	8 (17.0%)
○ 26-50%	22 (19.5%)	5 (9.6%)	0
○ 51-75%	7 (6.2%)	0	1 (2.1%)
○ 76-100%	15 (13.3%)	2 (3.8%)	4 (8.5%)
• Need for antivirals			
○ None	103 (91.1%)	50 (96.1%)	46 (97.9%)
○ 1-25%	9 (8.0%)	2 (3.8%)	1 (2.1%)
○ 26-50%	0	0	0
○ 51-75%	1 (0.9%)	0	0
○ 76-100%	0	0	0
• Need for anti-IL6 therapy			
○ None	111 (98.2%)	52 (100%)	47 (100%)
○ 1-25%	2 (1.8%)	0	0
○ 26-50%	0	0	0
○ 51-75%	0	0	0
○ 76-100%	0	0	0
• Need for hydroxychloroquine			
○ None	109 (96.5%)	51 (98.1%)	47 (100%)
○ 1-25%	3 (2.6%)	1 (1.9%)	0
○ 26-50%	1 (0.9%)	0	0
○ 51-75%	0	0	0
○ 76-100%	0	0	0
• Need for azithromycin			
○ None	86 (76.1%)	46 (88.5%)	43 (91.5%)
○ 1-25%	8 (7.1%)	3 (5.8%)	2 (4.3%)
○ 26-50%	10 (8.9%)	1 (1.9%)	1 (2.1%)
○ 51-75%	5 (4.4%)	2 (3.8%)	0
○ 76-100%	4 (3.5%)	0	1 (2.1%)
• Average glycemic control during the pandemic			
○ Mostly improved	21 (18.6%)	N/A	N/A
○ Mostly maintained same level	57 (50.4%)		
○ Mostly worsened	35 (31.0%)		

N/A, not applicable.

**Table 5:** Symptoms, complications, and outcomes of pediatric endocrine cases other than diabetes during the COVID-19 pandemic by clinical center.

	Obesity and Metabolic Syndrome (n=65)	Hyperinsulinemic hypoglycemia (n=41)	Thyroid disorders (n=91)	Adrenal disorders (n=66)	Bone metabolism disorders (n=32)	Pituitary and other CNS disorders (n=62)	Growth disorders (n=73)	Puberty disorders (n=69)
**Symptoms and/or complications among COVID-19 cases**								
• Asymptomatic								
○ None	40 (61.5%)	32 (78.0%)	57 (62.6%)	48 (72.7%)	24 (75.0%)	43 (69.3%)	46 (63.0%)	45 (65.2%)
○ 1-25%	5 (7.7%)	1 (2.5%)	8 (8.8%)	5 (7.6%)	1 (3.1%)	2 (3.2%)	3 (4.1%)	1 (1.4%)
○ 26-50%	7 (10.8%)	1 (2.5%)	5 (5.5%)	3 (4.5%)	1 (3.1%)	6 (9.7%)	8 (11.0%)	6 (8.7%)
○ 51-75%	4 (6.1%)	1 (2.5%)	8 (8.8%)	2 (3.0%)	1 (3.1%)	3 (4.8%)	4 (5.5%)	6 (8.7%)
○ 76-100%	9 (13.9%)	6 (14.6%)	13 (14.1%)	8 (12.1%)	5 (15.6%)	8 (12.9%)	12 (16.4%)	11 (15.9%)
• Fever								
○ None	36 (55.4%)	30 (73.2%)	61 (67.0%)	48 (72.7%)	25 (78.1%)	42 (67.7%)	48 (65.7%)	51 (73.9%)
○ 1-25%	12 (18.5%)	4 (9.8%)	18 (19.8%)	6 (9.1%)	1 (3.1%)	9 (14.5%)	15 (20.5%)	8 (11.6%)
○ 26-50%	7 (10.8%)	4 (9.8%)	7 (7.7%)	9 (13.6%)	4 (12.5%)	5 (8.1%)	8 (11.0%)	6 (8.7%)
○ 51-75%	7 (10.8%)	3 (7.3%)	5 (5.5%)	3 (4.5%)	2 (6.2%)	4 (6.4%)	2 (2.7%)	3 (4.3%)
○ 76-100%	3 (4.6%)	0	0	0	0	2 (3.2%)	0	1 (1.4%)
• Cough								
○ None	35 (53.8%)	31 (75.6%)	61 (67.0%)	50 (75.8%)	25 (78.1%)	42 (67.7%)	49 (67.1%)	50 (72.5%)
○ 1-25%	17 (26.1%)	5 (12.2%)	19 (20.9%)	9 (13.6%)	2 (6.2%)	10 (16.1%)	14 (19.2%)	10 (14.5%)
○ 26-50%	7 (10.8%)	2 (4.9%)	7 (7.7%)	5 (7.6%)	3 (9.4%)	6 (9.7%)	7 (9.6%)	5 (7.2%)
○ 51-75%	4 (6.1%)	2 (4.9%)	4 (4.4%)	2 (3.0%)	2 (6.2%)	2 (3.2%)	3 (4.1%)	3 (4.3%)
○ 76-100%	2 (3.1%)	1 (2.4%)	0	0	0	2 (3.2%)	0	1 (1.4%)
• Pharyngeal erythema								
○ None	44 (67.7%)	33 (80.5%)	70 (76.9%)	53 (80.3%)	27 (84.4%)	49 (79.0%)	53 (72.6%)	53 (76.8%)
○ 1-25%	13 (20.0%)	5 (12.2%)	14 (15.4%)	7 (10.6%)	2 (6.2%)	7 (11.3%)	13 (17.8%)	10 (14.5%)
○ 26-50%	2 (3.1%)	0	4 (4.4%)	4 (6.1%)	1 (3.1%)	4 (6.4%)	3 (4.1%)	4 (5.8%)
○ 51-75%	6 (9.2%)	2 (4.9%)	3 (3.3%)	2 (3.0%)	2 (6.2%)	2 (3.2%)	4 (5.5%)	2 (2.9%)
○ 76-100%	0	1 (2.4%)	0	0	0	0	0	0
• Rhinorrhea								
○ None	41 (63.1%)	30 (73.2%)	65 (71.4%)	49 (74.2%)	27 (84.4%)	43 (69.3%)	50 (68.5%)	52 (75.3%)
○ 1-25%	10 (15.4%)	6 (14.6%)	16 (17.6%)	9 (13.6)	2 (6.2%)	10 (16.1%)	14 (19.2%)	9 (13.0%)
○ 26-50%	7 (10.8%)	2 (4.9%)	5 (5.5%)	6 (9.1)	1 (3.1%)	5 (8.1%)	5 (6.8%)	4 (5.8%)
○ 51-75%	7 (10.8%)	2 (4.9%)	4 (4.4%)	2 (3.0%)	2 (6.2%)	3 (4.8%)	4 (5.5%)	4 (5.8%)
○ 76-100%	0	1 (2.4%)	1 (1.1%)	0	0	1 (1.6%)	0	0
• Shortness of breath								
○ None	48 (73.8%)	38 (92.7%)	79 (86.8%)	58 (87.9%)	26 (81.2%)	54 (87.1%)	65 (89.0%)	62 (89.9%)
○ 1-25%	12 (18.5%)	2 (4.9%)	11 (12.1%)	5 (7.6%)	4 (12.5%)	6 (9.7%)	6 (8.2%)	5 (7.2%)
○ 26-50%	3 (4.6%)	1 (2.4%)	1 (1.1%)	3 (4.5%)	2 (6.2%)	2 (3.2%)	1 (1.4%)	1 (1.4%)
○ 51-75%	2 (3.1%)	0	0	0	0	0	1 (1.4%)	0
○ 76-100%	0	0	0	0	0	0	0	1 (1.4%)
• Headache								
○ None	40 (61.5%)	34 (82.9%)	66 (72.5%)	53 (80.3%)	26 (81.2%)	47 (75.8%)	53 (72.6%)	51 (73.9%)
○ 1-25%	8 (12.3%)	4 (9.8%)	16 (17.6%)	6 (9.1%)	3 (9.4%)	7 (11.3%)	12 (16.4%)	9 (13.0%)
○ 26-50%	12 (18.5%)	0	6 (6.6%)	3 (4.5%)	2 (6.2%)	3 (4.8%)	4 (5.5%)	5 (7.2%)
○ 51-75%	5 (7.7%)	2 (4.9%)	3 (3.3%)	4 (6.1%)	1 (3.1%)	5 (8.1%)	4 (5.5%)	4 (5.8%)
○ 76-100%	0	1 (2.4%)	0	0	0	0	0	0
• Myalgia								
○ None	39 (60.0%)	34 (82.9%)	64 (70.3%)	53 (80.3%)	26 (81.2%)	46 (74.2%)	52 (71.2%)	51 (73.9%)
○ 1-25%	10 (15.4%)	6 (14.6%)	21 (23.1%)	7 (10.6%)	3 (9.4%)	8 (12.9%)	15 (20.5%)	13 (18.8%)
○ 26-50%	10 (15.4%)	0	3 (3.3%)	4 (6.1%)	2 (6.2%)	5 (8.1%)	4 (5.5%)	4 (5.8%)
○ 51-75%	6 (9.2%)	1 (2.4%)	3 (3.3%)	2 (3.0%)	1 (3.1%)	2 (3.2%)	2 (2.7%)	1 (1.4%)
○ 76-100%	0	0	0	0	0	1 (1.6%)	0	0
• Gastrointestinal pain								
○ None	39 (60.0%)	32 (78.0%)	63 (69.2%)	48 (72.7%)	26 (81.2%)	47 (75.8%)	52 (71.2%)	52 (75.4%)
○ 1-25%	12 (18.5%)	7 (17.1%)	19 (20.9%)	7 (10.6%)	4 (12.5%)	9 (14.5%)	16 (21.9%)	13 (18.8%)
○ 26-50%	7 (10.8%)	0	7 (7.7%)	8 (12.1%)	1 (3.1%)	4 (6.4%)	2 (2.7%)	2 (2.9%)
○ 51-75%	7 (10.8%)	2 (4.9%)	2 (2.2%)	3 (4.5%)	1 (3.1%)	2 (3.2%)	3 (4.1%)	2 (2.9%)
○ 76-100%	0	0	0	0	0	0	0	0
• Other specific	[Dysglycemia]	[Severe hypoglycemia]		[Adrenal crisis]				
○ None	53 (81.5%)	34 (82.9%)	N/A	58 (87.9%)	N/A	N/A	N/A	N/A
○ 1-25%	4 (6.1%)	4 (9.8%)		5 (7.6%)				
○ 26-50%	5 (7.7%)	1 (2.4%)		2 (3.0%)				
○ 51-75%	3 (4.6%)	0		1 (1.5%)				
○ 76-100%	0	2 (4.9%)		0				
**Outcomes among COVID-19 cases**								
• Admission								
○ None	54 (83.1%)	35 (85.4%)	86 (94.5%)	56 (84.8%)	28 (87.5%)	56 (90.3%)	69 (94.5%)	66 (95.6%)
○ 1-25%	10 (15.4%)	3 (7.3%)	4 (4.4%)	6 (9.1%)	3 (9.4%)	2 (3.2%)	3 (4.1%)	1 (1.4%)
○ 26-50%	0	0	0	0	0	2 (3.2%)	0	0
○ 51-75%	0	0	0	2 (3.0%)	0	0	0	0
○ 76-100%	1 (1.5%)	3 (7.3%)	1 (1.1%)	2 (3.0%)	1 (3.1%)	2 (3.2%)	1 (1.4%)	2 (2.9%)
• Admission to intensive care unit								
○ None	63 (96.9%)	40 (97.7%)	88 (96.7%)	64 (97.0%)	30 (93.8%)	60 (96.8%)	72 (98.6%)	68 (98.5%)
○ 1-25%	2 (3.1%)	1 (2.4%)	3 (3.3%)	1 (1.5)	2 (6.2%)	2 (3.2%)	1 (1.4%)	1 (1.5%)
○ 26-50%	0	0	0	1 (1.5%)	0	0	0	0
○ 51-75%	0	0	0	0	0	0	0	0
○ 76-100%	0	0	0	0	0	0	0	0
• Need for bronchodilators and glucocorticoids								
○ None	55 (84.6%)	40 (97.6%)	85 (93.4%)	62 (93.9%)	31 (96.9%)	59 (95.2%)	70 (95.9%)	69 (100%)
○ 1-25%	8 (12.3%)	1 (2.4%)	6 (6.6%)	2 (3.0%)	1 (3.1%)	2 (3.2%)	2 (2.7%)	0
○ 26-50%	0	0	0	1 (1.5%)	0	1 (1.6%)	1 (1.4%)	0
○ 51-75%	1 (1.5%)	0	0	0	0	0	0	0
○ 76-100%	1 (1.5%)	0	0	1 (1.5%)	0	0	0	0
• Need for oxygen								
○ None	58 (89.2%)	38 (92.7%)	86 (94.5%)	60 (90.9%)	29 (90.6%)	58 (93.6%)	70 (95.9%)	67 (97.1%)
○ 1-25%	6 (9.2%)	3 (7.3%)	5 (5.6%)	5 (7.6%)	3 (9.4%)	4 (6.4%)	2 (2.7%)	1 (1.4%)
○ 26-50%	0	0	0	1 (1.5%)	0	0	1 (1.4%)	0
○ 51-75%	1 (1.5%)	0	0	0	0	0	0	0
○ 76-100%	0	0	0	0	0	0	0	1 (1.4%)
• Need for non-invasive ventilation								
○ None	62 (95.4%)	38 (92.7%)	87 (95.6%)	64 (97.0%)	31 (96.9%)	60 (96.8%)	71 (97.3%)	68 (98.5%)
○ 1-25%	3 (4.6%)	3 (7.3%)	4 (4.4%)	2 (3.0%)	1 (3.1%)	2 (3.2%)	2 (2.7%)	1 (1.5%)
○ 26-50%	0	0	0	0	0	0	0	0
○ 51-75%	0	0	0	0	0	0	0	0
○ 76-100%	0	0	0	0	0	0	0	0
• Need for intubation and ventilation								
○ None	63 (96.9%)	40 (97.6%)	88 (96.7%)	65 (98.5%)	31 (96.9%)	61 (98.4%)	72 (98.6%)	69 (100%)
○ 1-25%	2 (3.1%)	0	3 (3.3%)	1 (1.5%)	0	1 (1.6%)	1 (1.4%)	0
○ 26-50%	0	1 (2.4%)	0	0	1 (3.1%)	0	0	0
○ 51-75%	0	0	0	0	0	0	0	0
○ 76-100%	0	0	0	0	0	0	0	0
• No need for specific treatments								
○ None	44 (67.7%)	33 (80.5%)	75 (82.4%)	56 (84.8%)	24 (75.0%)	50 (80.7%)	57 (78.1%)	55 (79.7%)
○ 1-25%	4 (6.1%)	1 (2.4%)	3 (3.3%)	2 (3.0%)	2 (6.2%)	2 (3.2%)	2 (2.7%)	1 (1.4%)
○ 26-50%	4 (6.1%)	0	4 (4.4%)	0	2 (6.2%)	3 (4.8%)	3 (4.1%)	3 (4.3%)
○ 51-75%	1 (1.5%)	1 (2.4%)	1 (1.1%)	2 (3.0%)	0	2 (3.2%)	2 (2.7%)	0
○ 76-100%	12 (18.5%)	6 (14.6%)	8 (8.8%)	6 (9.1%)	4 (12.5%)	5 (8.1%)	9 (12.3%)	10 (14.5%)
• Dose adjustment of background treatment								
○ None	50 (76.9%)	34 (82.9%)	75 (82.4%)	47 (71.2%)	27 (84.4%)	48 (77.4%)	64 (87.7%)	65 (94.2%)
○ 1-25%	11 (16.9%)	5 (12.2%)	12 (13.2%)	11 (16.7%)	3 (9.4%)	7 (11.3%)	6 (8.2%)	3 (4.3%)
○ 26-50%	1 (1.5%)	0	3 (3.3%)	2 (3.0%)	1 (3.1%)	3 (4.8%)	1 (1.4%)	0
○ 51-75%	1 (1.5%)	0	0	1 (1.5%)	0	2 (3.2%)	1 (1.4%)	0
○ 76-100%	2 (3.1%)	2 (4.9%)	1 (1.1%)	5 (7.6%)	1 (3.1%)	2 (3.2%)	1 (1.4%)	1 (1.4%)
• Need for antivirals								
○ None	63 (96.9%)	40 (97.6%)	90 (98.9%)	65 (98.5%)	31 (96.9%)	60 (96.8%)	71 (97.3%)	68 (98.5%)
○ 1-25%	2 (3.1%)	1 (2.4%)	1 (1.1%)	0	1 (3.1%)	2 (3.2%)	2 (2.7%)	1 (1.5%)
○ 26-50%	0	0	0	0	0	0	0	0
○ 51-75%	0	0	0	0	0	0	0	0
○ 76-100%	0	0	0	1 (1.5%)	0	0	0	0
• Need for anti-IL6 therapy								
○ None	65 (100%)	41 (100%)	91 (100%)	66 (100%)	32 (100%)	62 (100%)	73 (100%)	69 (100%)
○ 1-25%	0	0	0	0	0	0	0	0
○ 26-50%	0	0	0	0	0	0	0	0
○ 51-75%	0	0	0	0	0	0	0	0
○ 76-100%	0	0	0	0	0	0	0	0
• Need for hydroxychloroquine								
○ None	65 (100%)	41 (100%)	91 (100%)	66 (100%)	32 (100%)	62 (100%)	73 (100%)	69 (100%)
○ 1-25%	0	0	0	0	0	0	0	0
○ 26-50%	0	0	0	0	0	0	0	0
○ 51-75%	0	0	0	0	0	0	0	0
○ 76-100%	0	0	0	0	0	0	0	01
• Need for azithromycin								
○ None	50 (76.9%)	35 (85.4%)	79 (86.8%)	55 (83.3%)	28 (87.5%)	54 (87.1%)	65 (89.0%)	62 (89.9%)
○ 1-25%	8 (12.3%)	4 (9.8%)	9 (9.9%)	6 (9.1%)	1 (3.1%)	4 (6.5%)	5 (6.9%)	4 (5.8%)
○ 26-50%	7 (10.8%)	1 (2.4%)	3 (3.3%)	2 (3.0%)	2 (6.2%)	3 (4.8%)	1 (1.4%)	2 (2.9%)
○ 51-75%	0	0	0	0	0	0	1 (1.4%)	1 (1.4%)
○ 76-100%	0	1 (2.4%)	0	3 (4.5%)	1 (3.1%)	1 (1.6%)	1 (1.4%)	0

N/A, not applicable.

General therapeutic measures for COVID-19 were usually unnecessary. In most centers, patients did not need to be admitted in hospital, and most of did not need intensive care unit (ICU) beds, except for patients with diabetes (21.2%) (**Figure 1B**, **Tables 4**, **5**). The proportion of patients who needed bronchodilators and glucocorticoids more frequently was higher in type 1 diabetes (24.8%), type 2 diabetes (13.5%), and obesity (15.4%), than patients with other endocrine conditions tested positive for COVID-19. They also needed oxygen (28.3%), non-invasive ventilation (15.9%), intubation and ventilation (9.7%), and other therapies (e.g., antibiotics in 23.9%, antiviral agents in 8.9%) more often than in other endocrine conditions (**Tables 4**, **5**).

Although specific therapeutic measures for endocrine management were only infrequently needed in patients with COVID-19, background treatment dose adjustment was common: in 56.6% of centers, patients with type 1 diabetes needed insulin adjustments and in 26.9% of centers, patients with type 2 diabetes added insulin or adjusted oral medications. Similar adjustments to specific therapies were reported in 27.6% of centers for other forms of diabetes, 23.1% for obesity, 28.8% for adrenal disorders, and 26.6% for pituitary and other CNS disorders (**Tables 4**, **5**).

### 3.3 Disease-Related Outcomes From Pediatric Endocrine Disorders

#### 3.3.1 Diabetes (All Forms)

**Figure 2:**
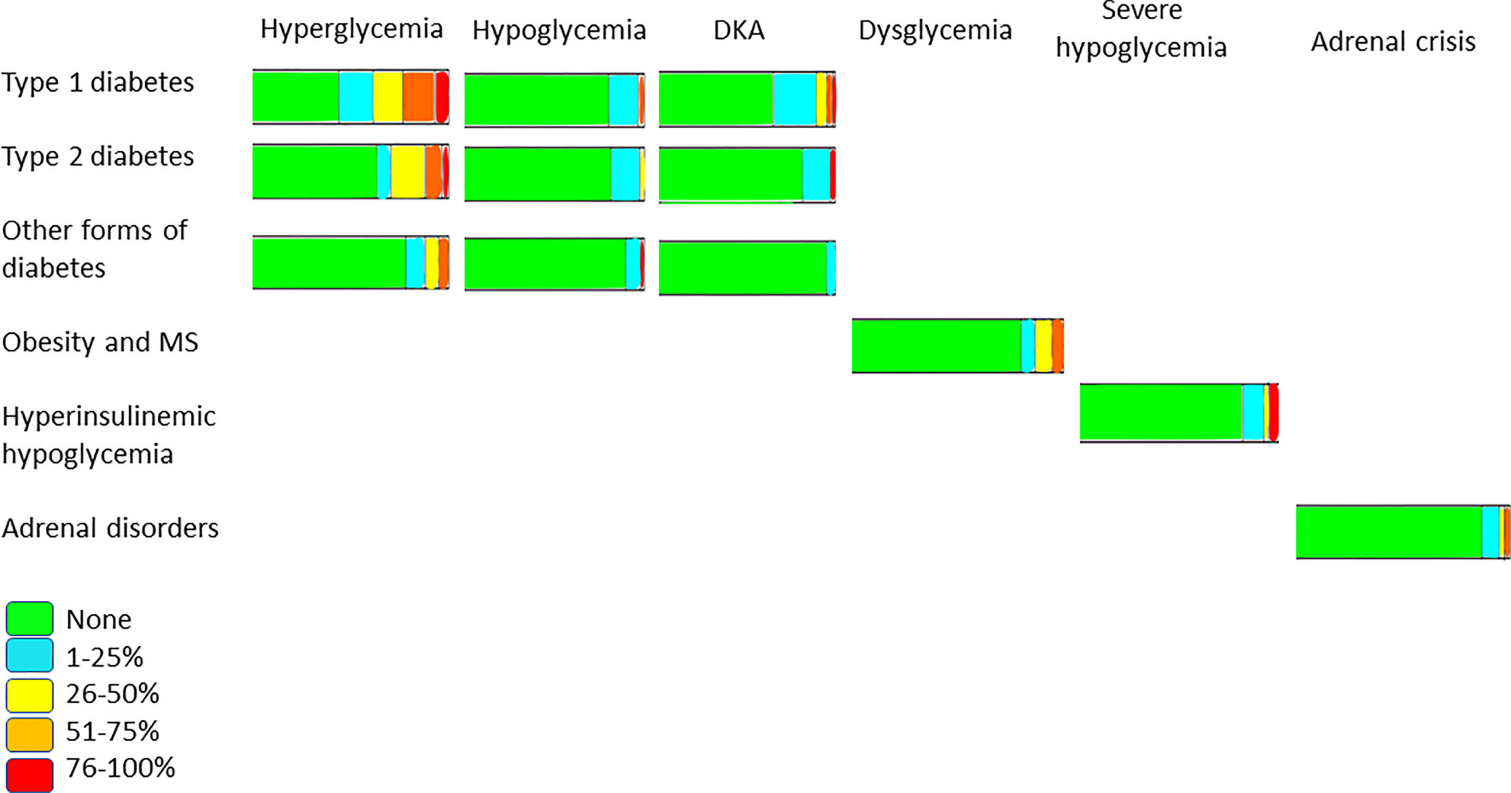
Proportion of specific symptoms in COVID-19 patients among pediatric endocrine centers.

Newly diagnosed mild to severe diabetic ketoacidosis (DKA) and new episodes in established cases were increased in 44% and 30% of centers, respectively (**Table 2**). In contrast, only 6% of centers reported severe hypoglycemia (**Figure 2**) .

Most HCPs performed COVID-19 testing in newly diagnosed patients (62.8%) and for DKA episodes in established patients (69.9%), which were negative in 49.6% of centers (**Table 2**).

#### 3.3.2 Other Endocrine Disorders

For most investigated endocrine disorders, there was some delay in new diagnoses as well as a worsening of the management. For patients who were diagnosed with COVID-19, most had asymptomatic or mild to moderate symptoms (**Tables 3**, **4** , **5**, **Figure 1**).

Remarkably, in patients with obesity or metabolic syndrome, ACE inhibitors, the most used antihypertensive drug (49.2%), were continued without interruption and with no complications reported to date (**Figure 1B**). Of note, in patients with HH, fewer than 10% reported at least one episode of severe hypoglycemia (**Figure 2**).

Two pediatric endocrine centers specialized in pediatric patients with gender dysphoria. Most patients were followed-up and educated by telephone consultation or F2F wearing appropriate PPE. In both centers, contact with the endocrine team was limited due the fear of the pandemic, and sometimes the diagnosis was possibly delayed but without perceived worsening of the management. Patients with gender dysphoria were not more likely to suffer from COVID-19, as seen for most other endocrine disorders.

### 3.4 Biopsychosocial and Behavior Outcomes From Pediatric Endocrine Disorders

In patients with type 1 diabetes, a lack of physical activity (41.6%) and poorer dietary choices (32.7%) with resulting increase in body weight (31.7%) were observed. Eighty percent of respondents reported high parental concerns about returning to school activities (**Table 2**). However, reassuringly, the majority (80%) had received specific school guidelines to keep children and teachers safe at school in the context of the COVID-19 pandemic. Moreover, general psychosocial and behavioral changes were commonly reported in patients with pediatric endocrine disorders including all forms of diabetes. The most commonly reported problems were anxiety, depression, parenting stress, sleep disruption, and eating disorders. Indeed, suicide attempts were seen in every condition except for puberty disorders (**Tables 2**, **3**).

## 4 Discussion

This survey shows that the proportion of patients with endocrine disorders testing positive for COVID-19 is low enough to not consider pediatric endocrine disorders as a poor prognostic factor for COVID-19. As previously reported, children and adolescents with any endocrine condition were not at increased risk of COVID-19 than children without endocrine conditions (15). However, comparing the proportion of patients with diabetes and COVID-19 in our previous survey with those reported here [only diabetes was assesses previously (9)], there was a significant increase in the number of children and adolescents with type 1 diabetes or other forms of diabetes who tested positive for COVID-19 (9). This is likely to be due to far more COVID-19 testing performed as the pandemic progressed compared to during the early phase of the pandemic (January-September 2020) and the reopening of schools in many countries.

While most pediatric patients with endocrine disorders affected by COVID-19 have asymptomatic or mild symptoms (16) it is important to note that patients with type 1 or type 2 diabetes were not only more likely to suffer from COVID-19 but also experience moderate to severe symptoms, especially when other comorbidities were present (17, 18). As a result, the number of patients with diabetes admitted to the ICU also increased compared to our previous survey (9), reaching a higher proportion of centers which reported admission for non-invasive ventilation (15.9%) and intubation and ventilation (9.7%) in comparison to other endocrine conditions in our survey (6% and 3%, respectively). Luckily, no deaths have been reported so far; diabetes and obesity are both risk factors for increased morbidity and mortality in adult patients with COVID-19 (19–21), but it is reassuring that in patients below 25 years the mortality rate approaches zero even when diabetic or obese.

While there are some data in adult patients (10–13), little is known about the impact of COVID-19 on other endocrine diseases in the pediatric population, and to our knowledge this is the first real-world and global study of this topic. The current experience of pediatric endocrinology HCPs suggests that the management of diabetes is much more demanding and complex than other endocrine disorders. There is a two-way association between COVID-19 and diabetes mellitus. In adults, uncontrolled diabetes is related to the severity of COVID-19. Moreover, severe metabolic complications, including DKA, have been observed in patients with COVID-19, either at onset (22, 23) or in pre-existing diabetes (24). While pediatric patients with diabetes do not appear to be at higher risk of infection by SARS-CoV-2, it is still prudent to avoid being infected and to adopt all preventive measures possible (25). It has also been hypothesized that SARS-CoV-2 might itself be diabetogenic, like in patients with SARS coronavirus 1 pneumonia (26). However, this association has yet to be confirmed and requires further study in both adults and children.

The present survey confirms that the COVID-19 pandemic delayed hospital admissions for diabetes and other endocrine diseases, resulting, for example, in a higher proportion of severe DKA cases, as observed elsewhere (22, 23, 27). A secure non-COVID-19 path through pediatric emergency departments is essential to help and reassure parents who wish to bring their children to hospital in a timely manner and avoid unnecessary complications in diabetes and other endocrine disorders (22).

Once endocrine disease is established, it is important to maintain an uninterrupted link with HCPs, as recommended by many endocrine societies (9, 28, 29), especially with telemedicine to avoid crowded waiting rooms. However, in the present survey, we observed that despite the need to minimize unnecessary hospital visits during the pandemic using dedicated platforms or video calls, text messaging, and emails, routine F2F visits remained the most common method of consultation. Improved knowledge of telemedicine might help families and patients gain trust in this way of delivering care. Prioritization of guidance on care management and accelerated innovation in telehealth is necessary to avoid complications in these patients, especially in limited resource settings. Video platforms have been implemented in many institutions, especially for education, although not all allow telehealth for inpatient care and the efficacy of telemedicine for education remains uncertain [30-32].

Many parents were concerned about the safety of sending their children with endocrine disorders, especially diabetes, back to school during the COVID-19 pandemic, believing that they had greater likelihood of contracting coronavirus. Reassuringly, however, the majority were familiar with school guidelines and ensured that a disease care plan was in place (25, 30, 31).

Ready access to endocrine and diabetes care medications and supplies, already a problem in large parts of the world before the pandemic, is of vital importance. With important infrastructure such as outpatient clinics and public transport severely limited by the pandemic, access issues have been exacerbated, even though daily self-management, sick day management, and survival depends on the availability of medical supplies. Fortunately, in the present survey, relatively few (6-22% according to different endocrine disorders) centers experienced a shortage of supplies.

We observed that COVID-19 was related to endocrine disease and comorbidities, with obesity and hypertension the most commonly reported in all endocrine diseases. Children and adolescents who needed intensive care often had comorbidities. Therefore, it is essential to understand which modifiable risk factors might play a role in increasing the severity of COVID-19 (32–36). Indeed, some children suffered from more serious COVID-19, but the reasons remain unclear; comorbidities are less frequent in young patients that in adults, which might explain why children are less vulnerable to COVID-19 but why some still fall critically ill. Given the recent rise in type 2 diabetes and obesity in youth, there could be a significant number of children at higher risk.

The COVID-19 pandemic has exacerbated mental health comorbidities throughout society, not least in patients with diabetes and other endocrine disorders (37, 38). Children are a particularly vulnerable group, as their nervous systems, endocrine systems, and hypothalamic-pituitary-adrenal axes are not well developed. Psychological crises often result in feelings of abandonment, despair, incapacity, and exhaustion in children, and even raise the risk of suicide. Of note, our survey revealed reports of suicide attempts during the pandemic in children with a wide range of different endocrine conditions. Psychosocial support for children and their families, especially those with chronic health conditions, must be a part of the health response to disaster and disaster recovery. Timely and appropriate protections are needed to prevent psychological and behavioral problems. Emerging digital applications and health services such as telehealth, social media, mobile health, and remote interactive online education may help bridge the social distance and support mental and behavioral health for children (39).

The SARS-CoV-2 virus has multiple pathophysiologic interconnections with endocrine systems with the potential to cause disturbances in pituitary, adrenal and thyroid function, and mineral metabolism. Data regarding the risks of SARS-CoV-2 infection in individuals with underlying endocrine disorders have primarily been described in adults (40). However, the limited existing data are generally favorable in terms of endocrine complications of COVID-19 in the pediatric population (41), as confirmed in our survey, where children with well-managed endocrine conditions did not seem to be at increased risk of getting infected or becoming severely ill with COVID-19.

Prior studies showed successful use of telemedicine for pediatric obesity, but it can be challenging to translate this process into telemedicine for other endocrine conditions (42).

Currently, there are no data indicating increased risk of acquiring SARS-CoV-2 infection or altered disease course in children and adolescents with underlying thyroid disorders. However, it is important to keep in mind that patients with Graves’ disease treated with anti-thyroid drug therapy are at higher risk of agranulocytosis and secondary infections (43). This is particularly important as data from one study showed that half of COVID-19 non-survivors experienced a secondary infection (44). Additionally, like other infections, COVID-19 may precipitate thyroid storm in patients with poorly controlled hyperthyroidism (41). Underlying thyroid disease, including hypothyroidism, does appear to be a risk factor for a more severe disease course in adults with COVID-19 (45–47). Children with metabolic bone disease or a skeletal dysplasia resulting in respiratory insufficiency due to altered chest wall structure may be at increased risk of COVID-19 complications (48).

Patients with primary adrenal insufficiency (e. g., congenital adrenal hyperplasia) are slightly more susceptible to infections in general, due to the impaired natural immunity function characterized by a defective action of neutrophils and natural killer cells, which is known to be associated with primary adrenal insufficiency (49). Furthermore, susceptibility to infections may also be explained by an insufficient increase of the hydrocortisone dosage at the beginning of an infection. Therefore, recommendations suggest that, if asymptomatic, children should remain on regular replacement doses of hydrocortisone and not increased doses. If symptoms suggestive of COVID-19, it is recommended to immediately increasing the hydrocortisone doses until the fever has subsided and adding an extra doubled dose.

Children diagnosed with hypopituitarism are also not at increased risk for COVID-19. As a significant percentage of these patients have secondary adrenal insufficiency, the same recommendations apply as for children with adrenal insufficiency (50). Hyperinsulinemic hypoglycemia side effects of the medications used to treat hyperinsulinemic hypoglycemia (e. g., diazoxide: water retention and pulmonary hypertension; somatostatin analogues: cardiac arrhythmias and cardiac conduction disorders) should be taken into consideration in the case of COVID-19. During this pandemic, children should follow management for hypoglycemia, which include close monitoring of glucose levels, adequate hydration, ensuring availability of medications and emergency regime. However, it is reassuring to note that survey data show that all these endocrine disorders did not cause any adjunctive distress to patients and the only serious issue to face is the possible shortage of medicines and/or supplies.

Majority of endocrine data are coming from recommendations issued by various health organizations and endocrine associations for the management of pediatric endocrine conditions during the pandemic. Adhering to the specific “sick day management rules” and undelayedly seeking for medical advice are only needed in most of the cases, as most children with endocrine disorders do not represent a high-risk population for contamination or severe presentation of COVID-19 (51).

Although it is difficult to analyze the effects of COVID-19 on endocrine disorders in children due to lack of studies and relatively less severe cases as compared to adults (52–54), looking at the data collected with the present survey, it appears that diabetes is still more challenging to manage than any other pediatric endocrine disorder with an increased risk of morbidity.

This study has some limitations. We received fewer overall responses than in the first survey, which focused on diabetes (51 vs. 75 countries and 131 endocrine centers vs. 215 diabetes centers, respectively). We hypothesize that HCPs perceive diabetes as more influenced by COVID-19 both directly because diabetes is a risk factor for COVID-19-related mortality and morbidity and indirectly because the pandemic can influence its management and supply availability. Other reasons for non-response might include workloads due to COVID-19, survey fatigue during the pandemic, insufficient reach of possible responders through email, perceived stress, and work-related burnout. Nevertheless, this is the first global study of the impact of COVID-19 in all pediatric endocrinology conditions. Most of the pediatric endocrinologists that responded to the survey were based in countries severely impacted by COVID-19 and worked in university/academic centers, strengthening the data and its global reach.

In conclusion, here we show that diabetes has been a particular management challenge during the COVID-19 pandemic and has an increased risk of morbidities including DKA. Specific strategies are essential to educate and reassure parents about maintaining close contact with their HCPs and about timely attendance at the emergency department when children develop symptoms unrelated to COVID-19. The global supply of essential medicines must always be maintained. Telemedicine requires strengthening and should be routine in every center. The mental health needs of children and adolescents with endocrine disorders must be addressed. International recommendations to reduce the severity of the impact of COVID-19 on pediatric patients with diabetes and other endocrine disorders must be devised with a special emphasis on its psychological impact.

## Data Availability Statement

The raw data supporting the conclusions of this article will be made available by the authors, without undue reservation.

## Author Contributions

NE drafted, revised, and approved the survey, analyzed data, drafted and discussed the manuscript. TS drafted, revised, and approved the survey, analyzed data, drafted and discussed the manuscript. CB drafted, revised, and approved the survey, and discussed the manuscript. EW, drafted, revised, and approved the survey, and discussed the manuscript. AP, drafted, revised, and approved the survey, and discussed the manuscript. AS drafted, revised, and approved the survey, drafted and discussed the manuscript. All authors contributed to the article and approved the submitted version.

## Conflict of Interest

The authors declare that the research was conducted in the absence of any commercial or financial relationships that could be construed as a potential conflict of interest.

## Publisher’s Note

All claims expressed in this article are solely those of the authors and do not necessarily represent those of their affiliated organizations, or those of the publisher, the editors and the reviewers. Any product that may be evaluated in this article, or claim that may be made by its manufacturer, is not guaranteed or endorsed by the publisher.
