# Supplementary material for: The Challenges of Managing Pediatric Diabetes and Other Endocrine Disorders During the COVID-19 Pandemic: Results From an International Cross-Sectional Electronic Survey
Source: Front Endocrinol (Lausanne). 2021 Nov 5;12:735554. doi: 10.3389/fendo.2021.735554 (PMC8602836; doi:10.3389/fendo.2021.735554)
Supplement: Supplementary file 1 [file DataSheet_1.pdf]

COVID-19 outbreak and pediatric endocrine disorders

Questions Responses

Section 1 of 29

COVID-19 outbreak and pediatric endocrine

Several measures used to contain COVID-19 pandemics like social distancing and lockdowns have also

Image title

After section 1 Continue to next section

Section 2 of 29

Your agreement to participate in this survey

Description (optional)

Do you agree to participate in this survey? *

- Yes
- No

After section 2 Continue to next section

Section 3 of 29

Healthcare professional profile

Description (optional)

Which country do you practice in? *

1. Afghanistan
2. Akrotiri
3. Albania
4. Algeria
5. American Samoa
6. Andorra
7. Angola
8. Anguilla
9. Antarctica
10. Antigua and Barbuda
11. Argentina
12. Armenia
13. Aruba
14. Ashmore and Cartier Islands
15. Australia

16. Austria
17. Azerbaijan
18. Bahamas, The
19. Bahrain
20. Bangladesh
21. Barbados
22. Bassas da India
23. Belarus
24. Belgium
25. Belize
26. Benin
27. Bermuda
28. Bhutan
29. Bolivia
30. Bosnia and Herzegovina
31. Botswana
32. Bouvet Island
33. Brazil
34. British Indian Ocean Territory
35. British Virgin Islands
36. [illegible]

36. Brunei
37. Bulgaria
38. Burkina Faso
39. Burma
40. Burundi
41. Cambodia
42. Cameroon
43. Canada
44. Cape Verde
45. Cayman Islands
46. Central African Republic
47. Chad
48. Chile
49. China
50. Christmas Island
51. Clipperton Island
52. Cocos (Keeling) Islands
53. Colombia
54. Comoros
55. Congo, Democratic Republic of the
56. Congo, Republic of the

57. Cook Islands
58. Coral Sea Islands
59. Costa Rica
60. Cote d'Ivoire
61. Croatia
62. Cuba
63. Cyprus
64. Czech Republic
65. Denmark
66. Dhekelia
67. Djibouti
68. Dominica
69. Dominican Republic
70. Ecuador
71. Egypt
72. El Salvador
73. Equatorial Guinea
74. Eritrea
75. Estonia
76. Ethiopia
77. Europa Island

78. Falkland Islands (Islas Malvinas)
79. Faroe Islands
80. Fiji
81. Finland
82. France
83. French Guiana
84. French Polynesia
85. French Southern and Antarctic Lands
86. Gabon
87. Gambia, The
88. Gaza Strip
89. Georgia
90. Germany
91. Ghana
92. Gibraltar
93. Glorioso Islands
94. Greece
95. Greenland
96. Grenada
97. Guadeloupe
98. Guam

99. Guatemala

100. Guernsey

101. Guinea

102. Guinea-Bissau

103. Guyana

104. Haiti

105. Heard Island and McDonald Islands

106. Holy See (Vatican City)

107. Honduras

108. Hong Kong

109. Hungary

110. Iceland

111. India

112. Indonesia

113. Iran

114. Iraq

115. Ireland

116. Isle of Man

117. Israel

118. Italy

[illegible]

119. Jamaica
120. Jan Mayen
121. Japan
122. Jersey
123. Jordan
124. Juan de Nova Island
125. Kazakhstan
126. Kenya
127. Kiribati
128. Korea, North
129. Korea, South
130. Kuwait
131. Kyrgyzstan
132. Laos
133. Latvia
134. Lebanon
135. Lesotho
136. Liberia
137. Libya
138. Liechtenstein
139. Lithuania

140. Luxembourg
141. Macau
142. Macedonia
143. Madagascar
144. Malawi
145. Malaysia
146. Maldives
147. Mali
148. Malta
149. Marshall Islands
150. Martinique
151. Mauritania
152. Mauritius
153. Mayotte
154. Mexico
155. Micronesia, Federated States of
156. Moldova
157. Monaco
158. Mongolia
159. Montserrat
160. Morocco

161. Mozambique
162. Namibia
163. Nauru
164. Navassa Island
165. Nepal
166. Netherlands
167. Netherlands Antilles
168. New Caledonia
169. New Zealand
170. Nicaragua
171. Niger
172. Nigeria
173. Niue
174. Norfolk Island
175. Northern Mariana Islands
176. Norway
177. Oman
178. Pakistan
179. Palau
180. Panama
181. Papua New Guinea

181. Papua New Guinea
182. Paracel Islands
183. Paraguay
184. Peru
185. Philippines
186. Pitcairn Islands
187. Poland
188. Portugal
189. Puerto Rico
190. Qatar
191. Reunion
192. Romania
193. Russia
194. Rwanda
195. Saint Helena
196. Saint Kitts and Nevis
197. Saint Lucia
198. Saint Pierre and Miquelon
199. Saint Vincent and the Grenadines
200. Samoa
201. San Marino

202. Sao Tome and Principe
203. Saudi Arabia
204. Senegal
205. Serbia and Montenegro
206. Seychelles
207. Sierra Leone
208. Singapore
209. Slovakia
210. Slovenia
211. Solomon Islands
212. Somalia
213. South Africa
214. South Georgia and the South Sandwich Islands
215. Spain
216. Spratly Islands
217. Sri Lanka
218. Sudan
219. Suriname
220. Svalbard
221. Swaziland
222. Sweden

223. Switzerland
224. Syria
225. Taiwan
226. Tajikistan
227. Tanzania
228. Thailand
229. Timor-Leste
230. Togo
231. Tokelau
232. Tonga
233. Trinidad and Tobago
234. Tromelin Island
235. Tunisia
236. Turkey
237. Turkmenistan
238. Turks and Caicos Islands
239. Tuvalu
240. Uganda
241. Ukraine
242. United Arab Emirates
243. United Kingdom

244. United States
245. Uruguay
246. Uzbekistan
247. Vanuatu
248. Venezuela
249. Vietnam
250. Virgin Islands
251. Wake Island
252. Wallis and Futuna
253. West Bank
254. Western Sahara
255. Yemen
256. Zambia
257. Zimbabwe

Kindly provide us with the name of your center and city/region to avoid duplicate responses *

Short answer text

What is your current clinical role? *

- ◯ Pediatric endocrinologist/diabetologist
- ◯ [illegible]

- ◯ Pediatrician with interest in endocrinology diseases and/or diabetes
- ◯ Adult physician looking after pediatric or adolescent patients
- ◯ Primary care practitioner/family doctor with interest in endocrinology diseases and/or diabetes
- ◯ Trainee/ fellow
- ◯ Researcher
- ◯ Nurse practitioner/registered nurse
- ◯ Dietitian
- ◯ Mental health professional
- ◯ Diabetes educator
- ◯ Other...

Where is your main practice setting? *

- ◯ Private hospital/clinic
- ◯ Public/governmental hospital/clinic
- ◯ University/Academic hospital/clinic
- ◯ Primary care center
- ◯ General practitioner office
- ◯ Other...

Have you taking care directly or indirectly of any child diagnosed with COVID-19? *

- ◯ Yes

No

Which method was (were) used to confirm COVID-19 diagnosis? *

RT-PCR

Antigenic tests

Serologic tests

Disease was not confirmed with a standard test but was assumed due to high clinical suspicion

No patient has been diagnosed so far

Did you fill the previous ISPAD survey entitled "COVID-19 outbreak and pediatric diabetes: perceptions of *

Yes

No

After section 3 Continue to next section

Section 4 of 29

Second assessment of previous ISPAD survey

If you were a participant of the ISPAD Survey entitled "COVID-19 outbreak and pediatric diabetes:

Image title

Estimated number of patients with diabetes affected since the beginning of COVID-19 outbreak *

Short answer text

Since the first survey how many patients were COVID-19 positive? *

Short answer text

Since the first survey, has COVID-19 influenced your daily routine visiting patients? *

- Yes
- No

If yes, how? *

- By using more often telemedicine
- By telemonitoring more often
- I have had difficulties in checking patient's glycemia because of technological problems
- COVID-19 has not influenced it
- Other...

In your country/region, how are children and adolescents mostly receiving education? *

- In-person
- Mixed in-person and virtually

- Virtually through distance learning tools
- Homeschooling (supervised by parents/caregivers)
- Not receiving yet because of lockdowns/social restrictions and no plan for school reopening

If you had any patient COVID-19 tested positive with a standard test, please estimate the number of those who

Short answer text

If you had any patient COVID-19 tested positive with a standard test, please estimate the number of those who

Short answer text

If you had any patient COVID-19 tested positive with a standard test, please estimate the number of those who

Short answer text

If you had any patient COVID-19 tested positive with a standard test, please estimate the number of those who

Short answer text

If you had any patient COVID-19 tested positive with a standard test, please estimate the number of those who

Short answer text

If you had any patient COVID-19 tested positive with a standard test, please estimate the number of those who

Short answer text

If you have any patient COVID-19 not tested due to limited resources but with a high clinical suspicion that

Short answer text

If you have any patient COVID-19 not tested due to limited resources but with a high clinical suspicion that

Short answer text

If you have any patient COVID-19 not tested due to limited resources but with a high clinical suspicion that

Short answer text

If you have any patient COVID-19 not tested due to limited resources but with a high clinical suspicion that

Short answer text

If you have any patient COVID-19 not tested due to limited resources but with a high clinical suspicion that

Short answer text

Do you have any national guidelines for children and adolescents at school during COVID-19 time? *

- Yes
- No

Do in your country local authorities impose protective social measures, such as: *

- [ ] Social distancing
- [ ] Facial mask inside
- [ ] Facial mask outside
- [ ] Lockdowns
- [ ] No restricted measures were mandatory
- [ ] Other...

Did your local Endocrinology/Diabetology societies develop specific guidelines for pediatric diabetes and *

- ( ) Yes
- ( ) No

Have you leafed through ISPAD and ESPE guidelines for pediatric diabetes and endocrine diseases and *

- ( ) Yes
- ( ) No

After section 4 Continue to next section

Section 5 of 29

Type 1 diabetes

Description (optional)

Do you look after children and young people with type 1 diabetes and want to answer to the questions *

- Yes
- No

After section 5 Continue to next section

Section 6 of 29

Type 1 diabetes

Description (optional)

How many children and young people aged 0-18 years with type 1 diabetes do you look after? *

- Less than 100
- 101 to 250
- 251 to 500
- More than 500

How do you maintain routine checkup effectively and efficiently during COVID-19 outbreak? *

- [ ] As usual, no changes
- [ ] Sent SMS and emails for consultations
- [ ] Telephone consultations

- [ ] Video consultations
- [ ] Apps
- [ ] Face to face consultation with appropriate personal protective equipment restricted to just one parent/caregiver
- [ ] Face to face consultation with appropriate personal protective equipment where all caregivers are allowed to attend
- [ ] Other…

Have you had any patient whose diagnose was delayed due to COVID-19 outbreak? *

- ( ) Yes
- ( ) No

Have the percentage of diabetic ketoacidosis (DKA) in new-onset cases increased in your practice during *

- ( ) Yes
- ( ) No

What was the proportion of severity of DKA? *

	0-25%	26-50%	51-75%	76-100%
Mild (pH < 7.3)	( )	( )	( )	( )
Moderate (pH < 7.2)	( )	( )	( )	( )
Severe (pH < 7.1)	( )	( )	( )	( )
No DKA	( )	( )	( )	( )

Did you perform RT-PCR or antigen test for those who are newly diagnosed diabetes? *

- Yes
- No

If yes, what is the percentage for those who were COVID -19 positive? *

- Less than 25%
- 26-50%
- 51-75%
- More than 75%
- No positivity with standardized tests

Have the percentage of DKA in established cases increased in your practice? *

- Yes
- No

Did you perform RT-PCR or antigen test for those who presented with DKA? *

- Yes
- No

If yes, what is the percentage for those who were tested COVID -19 positive?

- Less than 10%
- 10-25%
- 26-50%
- 51-75%
- More than 75%

Have you had the perception that DKA episodes have worsened since COVID-19 outbreak? *

- Yes
- No

Have the percentage of severe hypoglycemia episodes in patients with established diabetes increased in your *

- Yes
- No

How does your multidisciplinary team deliver education to patients with new onset type 1 diabetes during *

- Face to face education wearing appropriate personal protective equipment
- By telephone
- Video consultation
- Via Apps or digital plataforms
- As usual, no changes
- Other...

What is the refill prescription period in your region? *

- Refill prescription every month
- Refill prescription every 3 months or less
- Refill prescription every 6 months or less
- Refill prescription every year or less
- Automatic refill prescription from pharmacy
- I am not directly involved with prescription
- As required by patient

Have any of your patients had shortage of any diabetes medical supplies? *

- Yes
- No, everything was secured
- I am not aware of situation

If yes, what was (were) missing? *

- Basal insulin
- Bolus insulin
- Glucose test strips
- Blood glucose sensors
- Insulin pump supplies

- [ ] Ketone strips
- [ ] No shortage of any supply
- [ ] Other...

Do you have the feeling that parents/families avoid contact with diabetes team because of COVID-19 fear? *

- ◯ Yes
- ◯ No

If any of children and young people with type 1 diabetes followed by you was tested positive for COVID-19,

Short answer text

If any of children and young people with type 1 diabetes followed by you was tested positive for COVID-19,

Short answer text

If any of children and young people with type 1 diabetes followed by you was tested positive for COVID-19,

Short answer text

If any of children and young people with type 1 diabetes followed by you was tested positive for COVID-19,

Short answer text

If any of children and young people with type 1 diabetes followed by you was tested positive for COVID-19,

Short answer text

Do your patients have other comorbidities? *

- [ ] Asthma
- [ ] Cystic fibrosis or broncodisplasia
- [ ] Heart disease
- [ ] Kidney disease
- [ ] Cancer
- [ ] Obesity
- [ ] Hyperthension
- [ ] Other...

What was the mean proportion of patients with COVID-19 that exhibited the following? *

	0%	1-25%	26-50%	51-75%	76-100%
Fever	○	○	○	○	○
Cough	○	○	○	○	○
Pharyngeal eryt...	○	○	○	○	○
Rhinorrhea	○	○	○	○	○
Shortness of br...	○	○	○	○	○
Headache	○	○	○	○	○

Myalgia	○	○	○	○	○
Gastrointestina...	○	○	○	○	○
Hyperglycemia	○	○	○	○	○
Diabetic ketoac...	○	○	○	○	○
Hypoglycemia	○	○	○	○	○
Asymptomatic	○	○	○	○	○

What was the mean proportion of your patients with COVID-19 who needed the following? *

	0%	1-25%	26-50%	51-75%	76-100%
Admission to h...	○	○	○	○	○
Admission to In...	○	○	○	○	○
Bronchodilator...	○	○	○	○	○
Oxygen	○	○	○	○	○
Noninvasive ve...	○	○	○	○	○
Intubation and ...	○	○	○	○	○
No specific tre...	○	○	○	○	○

What was the mean proportion of your patients with COVID-19 who needed the following specific *

	0%	1-25%	26-50%	51-75%	76-100%
	○	○	○	○	○

Increased dose...					
Antivirals (Rem...	○	○	○	○	○
Anti IL-6	○	○	○	○	○
Hydroxychloroq...	○	○	○	○	○
Azithromicin	○	○	○	○	○

What advice have you recommended to your patients with regards to blood or sensor glucose monitoring? *

- [ ] No changes from usual practice
- [ ] Monitor blood glucose more frequently
- [ ] Monitor ketone strips more frequently
- [ ] Change to continuous glucose monitoring (CGM), when available
- [ ] Review CGM data more frequently
- [ ] Monitor blood glucose and review CGM data more frequently
- [ ] Other...

Have you seen psychological problems among children and young people with diabetes and their caregivers *

- [ ] None have had psychological problems so far
- [ ] Depression
- [ ] Panic attacks
- [ ] Anxiety
- [ ] Eating disorder

- [ ] Parenting stress
- [ ] Insomnia/hypersomnia
- [ ] Night terror
- [ ] Sleep disruption
- [ ] Suicide attempt
- [ ] Patient or caregivers have improved the mood
- [ ] Other...

For established cases with diabetes, during quarantine period what happened to glycemic control? *

- ○ Mostly improved glycemic control
- ○ Mostly worsened glycemic control
- ○ Mostly maintained same level of glycemic control

What is the average percentage of patients in your center who: *

	Less than 10%	10-25%	26-50%	51-75%	76-100%
Are on insulin p...	○	○	○	○	○
Are on continu...	○	○	○	○	○
Are on flash glu...	○	○	○	○	○
Maintained phy...	○	○	○	○	○
Faced worseni...	○	○	○	○	○

Increased body...

Do you feel that parents are afraid of returning to school and its impact upon safety of children with *

- Yes
- No

Have the schools in your region created specific guidelines for children during COVID-19 pandemic? *

- Yes
- No

After section 6 Continue to next section

Section 7 of 29

Type 2 diabetes

Description (optional)

Do you look after children and young people with type 2 diabetes and want to answer to the questions *

- Yes
- No

After section 7 Continue to next section

Section 8 of 29

Type 2 diabetes

Description (optional)

What is the estimated number of children and young people aged 0-18 years with type 2 diabetes do you *

Short answer text

How do you maintain routine checkup effectively and efficiently during COVID-19 outbreak? *

- [ ] As usual, no changes
- [ ] Sent SMS and emails for consultations
- [ ] Telephone consultations
- [ ] Video consultations
- [ ] Apps
- [ ] Face to face consultation with appropriate personal protective equipment
- [ ] Other...

Have you had any patient whose diagnose was delayed due to COVID-19 outbreak? *

- ○ Yes
- ○ No

If any of children and young people with type 2 diabetes followed by you was tested positive for COVID-19,

Short answer text

If any of children and young people with type 2 diabetes followed by you was tested positive for COVID-19,

Short answer text

If any of children and young people with type 2 diabetes followed by you was tested positive for COVID-19,

Short answer text

If any of children and young people with type 2 diabetes followed by you was tested positive for COVID-19,

Short answer text

If any of children and young people with type 2 diabetes followed by you was tested positive for COVID-19,

Short answer text

If any of children and young people with type 2 diabetes followed by you was tested positive for COVID-19,

Short answer text

Have you had the perception that glycemic control has worsened since COVID-19 outbreak? *

- Yes
- No

Did your patients need extra dose of insulin to control diabetes *

- ◯ Yes
- ◯ No
- ◯ Patient is not on insulin treatment

How does your multidisciplinary team deliver education to your patients during the outbreak? *

- ☐ Face to face education wearing appropriate personal protective equipment
- ☐ By telephone
- ☐ Video consultation
- ☐ Via Apps or digital plataforms
- ☐ As usual, no changes
- ☐ Other...

Have any of your patients had shortage of any diabetes medical supplies? *

- ◯ Yes
- ◯ No, everything was secured
- ◯ I am not aware of situation

If your patients have had shortage of any medical supply and/or diagnostic tests, please, state here:

Short answer text

Do you have the feeling that parents/families avoid contact with diabetes team because of COVID-19 fear? *

- Yes
- No

Do your patients have other comorbidities? *

- Asthma
- Cystic fibrosis or broncodisplasia
- Heart disease
- Kidney disease
- Cancer
- Obesity
- Hypertension
- Other...

In the case you have any patient with hypertension, what type of antihypertensive is mostly used to control blood

Short answer text

What was the mean proportion of patients with COVID-19 that exhibited the following? *

	0	1-25%	26-50%	51-75%	76-100%

Fever	○	○	○	○	○
Cough	○	○	○	○	○
Pharyngeal eryt...	○	○	○	○	○
Rhinorrhea	○	○	○	○	○
Shortness of br...	○	○	○	○	○
Headache	○	○	○	○	○
Myalgia	○	○	○	○	○
Gastrointestina...	○	○	○	○	○
Hyperglycemia	○	○	○	○	○
Diabetic ketoac...	○	○	○	○	○
Hypoglycemia	○	○	○	○	○
Asymptomatic	○	○	○	○	○

What was the mean proportion of your patients with COVID-19 who needed the following? *

	0%	1-25%	26-50%	51-75%	76-100%
Admission to h...	○	○	○	○	○
Admission to In...	○	○	○	○	○
Bronchodilator...	○	○	○	○	○
Oxygen	○	○	○	○	○

Noninvasive ve…	○	○	○	○	○
Intubation and …	○	○	○	○	○
No specific tre…	○	○	○	○	○

What was the mean proportion of your patients with COVID-19 who needed the following specific *

	0%	1-25%	26-50%	51-75%	76-100%
Increased dose…	○	○	○	○	○
Antivirals (Rem…	○	○	○	○	○
Anti IL-6	○	○	○	○	○
Hydroxychloroq…	○	○	○	○	○
Azithromicin	○	○	○	○	○

Have you seen psychological problems among children and young people with diabetes and their caregivers *

- [ ] None have had psychological problems so far
- [ ] Depression
- [ ] Panic attacks
- [ ] Anxiety
- [ ] Eating disorder
- [ ] Parenting stress
- [ ] Insomnia/hypersomnia
- [ ] [illegible]

- [ ] Night terror
- [ ] Sleep disruption
- [ ] Suicide attempt
- [ ] Patient or caregivers have improved the mood
- [ ] Other...

After section 8 Continue to next section

Section 9 of 29

Other forms of diabetes

Description (optional)

Do you look after children and young people with other forms of diabetes and want to answer to the *

- ( ) Yes
- ( ) No

After section 9 Continue to next section

Section 10 of 29

Other forms of diabetes

Description (optional)

What is the estimated number of children and young people aged 0-18 years with other forms of diabetes do *

Short answer text

How do you maintain routine checkup effectively and efficiently during COVID-19 outbreak? *

- [ ] As usual, no changes
- [ ] Sent SMS and emails for consultations
- [ ] Telephone consultations
- [ ] Video consultations
- [ ] Apps
- [ ] Face to face consultation with appropriate personal protective equipment
- [ ] Other...

Have you had any patient whose diagnose was delayed due to COVID-19 outbreak? *

- ( ) Yes
- ( ) No

Have you had the perception that glycemic control has worsened since COVID-19 outbreak? *

- ( ) Yes
- ( ) No

How does your multidisciplinary team deliver education to your patients during the outbreak? *

- [ ] Face to face education wearing appropriate personal protective equipment
- [ ] By telephone
- [ ] Video consultation
- [ ] Via Apps or digital plataforms
- [ ] As usual, no changes
- [ ] Other…

Have any of your patients had shortage of any medical supplies? *

- ◯ Yes
- ◯ No, everything was secured
- ◯ I am not aware of situation

If your patients have had shortage of any medical supply and/or diagnostic tests, please, state here:

Short answer text

Do you have the feeling that parents/families avoid contact with diabetes team because of COVID-19 fear? *

- ◯ Yes
- ◯ No

Do your patients have other comorbidities? *

- [ ] Asthma

- [ ] Cystic fibrosis or broncodisplasia
- [ ] Heart disease
- [ ] Kidney disease
- [ ] Cancer
- [ ] Obesity
- [ ] Hypertension
- [ ] Other...

What was the mean proportion of patients with COVID-19 that exhibited the following? *

	0	1-25%	26-50%	51-75%	76-100%
Fever	○	○	○	○	○
Cough	○	○	○	○	○
Pharyngeal eryt...	○	○	○	○	○
Rhinorrhea	○	○	○	○	○
Shortness of br...	○	○	○	○	○
Headache	○	○	○	○	○
Myalgia	○	○	○	○	○
Gastrointestina...	○	○	○	○	○
Hyperglycemia	○	○	○	○	○
Diabetic ketoac...	○	○	○	○	○

	0%	1-25%	26-50%	51-75%	76-100%
Hypoglycemia	○	○	○	○	○
Asymptomatic	○	○	○	○	○

What was the mean proportion of your patients with COVID-19 who needed the following? *

	0%	1-25%	26-50%	51-75%	76-100%
Admission to h...	○	○	○	○	○
Admission to In...	○	○	○	○	○
Bronchodilator...	○	○	○	○	○
Oxygen	○	○	○	○	○
Noninvasive ve...	○	○	○	○	○
Intubation and ...	○	○	○	○	○
No specific tre...	○	○	○	○	○

What was the mean proportion of your patients with COVID-19 who needed the following specific *

	0%	1-25%	26-50%	51-75%	76-100%
Dose adjustme...	○	○	○	○	○
Antiviral (Remd...	○	○	○	○	○
Anti IL-6	○	○	○	○	○
Hydroxychloroq...	○	○	○	○	○
Azithromicin	○	○	○	○	○

Have you seen psychological problems among children and young people with diabetes and their caregivers *

- [ ] None have had psychological problems so far
- [ ] Depression
- [ ] Panic attacks
- [ ] Anxiety
- [ ] Eating disorder
- [ ] Parenting stress
- [ ] Insomnia/hypersomnia
- [ ] Night terror
- [ ] Sleep disruption
- [ ] Suicide attempt
- [ ] Patient or caregivers have improved the mood
- [ ] Other...

After section 10 Continue to next section

Section 11 of 29

Obesity and Metabolic Syndrome

Description (optional)

Do you look after children and young people with obesity and metabolic syndrome and want to answer to *

- ◯ Yes
- ◯ No

After section 11 Continue to next section

Section 12 of 29

Obesity and Metabolic syndrome

Description (optional)

What is the estimated number of children and young people aged 0-18 years with obesity and/or metabolic *

Short answer text

How do you maintain routine checkup effectively and efficiently during COVID-19 outbreak? *

- ☐ As usual, no changes
- ☐ Sent SMS and emails for consultations
- ☐ Telephone consultations
- ☐ Video consultations
- ☐ Apps
- ☐ Face to face consultation with appropriate personal protective equipment
- ☐ Other...

Have you had any patient whose diagnose was delayed due to COVID-19 outbreak? *

- Yes
- No

Have you had the perception that disease management has worsened since COVID-19 outbreak? *

- Yes
- No

How does your multidisciplinary team deliver education to your patients during the outbreak? *

- [ ] Face to face education wearing appropriate personal protective equipment
- [ ] By telephone
- [ ] Video consultation
- [ ] Via Apps or digital plataforms
- [ ] As usual, no changes
- [ ] Other...

Have any of your patients had shortage of any medical supplies? *

- Yes
- No, everything was secured
- I am not aware of situation

If your patients have had shortage of any medical supply and/or diagnostic tests, please, state here:

Short answer text

Do you have the feeling that parents/families avoid contact with endocrine team because of COVID-19 fear? *

- Yes
- No

Do your patients have other comorbidities? *

- Asthma
- Cystic fibrosis or broncodisplasia
- Heart disease
- Kidney disease
- Cancer
- Hyperthension
- Other...

In the case you have any patient with hypertension, what type of antihypertensive is mostly used to control blood

Short answer text

Did you advice to continue using it during COVID-19? *

- Yes
- No
- No patient on antihypertensives

Have you seen any complication due to its use? *

- Yes
- No
- No patient on antihypertensives

What was the mean proportion of patients with COVID-19 that exhibited the following? *

	0	1-25%	26-50%	51-75%	76-100%
Fever	○	○	○	○	○
Cough	○	○	○	○	○
Pharyngeal eryt...	○	○	○	○	○
Rhinorrhea	○	○	○	○	○
Shortness of br...	○	○	○	○	○
Headache	○	○	○	○	○
Myalgia	○	○	○	○	○
Gastrointestina...	○	○	○	○	○
Glucose flutuat...	○	○	○	○	○

Asymptomatic	◯	◯	◯	◯	◯

What was the mean proportion of your patients with COVID-19 who needed the following? *

	0%	1-25%	26-50%	51-75%	76-100%
Admission to h...	◯	◯	◯	◯	◯
Admission to In...	◯	◯	◯	◯	◯
Bronchodilator...	◯	◯	◯	◯	◯
Oxygen	◯	◯	◯	◯	◯
Noninvasive ve...	◯	◯	◯	◯	◯
Intubation and ...	◯	◯	◯	◯	◯
No specific tre...	◯	◯	◯	◯	◯

What was the mean proportion of your patients with COVID-19 who needed the following specific *

	0%	1-25%	26-50%	51-75%	76-100%
Dose adjustme...	◯	◯	◯	◯	◯
Antivirals (Rem...	◯	◯	◯	◯	◯
Anti IL-6	◯	◯	◯	◯	◯
Hydroxychloroq...	◯	◯	◯	◯	◯
Azithromicin	◯	◯	◯	◯	◯

Have you seen psychological problems among children and young people with obesity/metabolic syndrome *

- [ ] None have had psychological problems so far
- [ ] Depression
- [ ] Panic attacks
- [ ] Anxiety
- [ ] Eating disorder
- [ ] Parenting stress
- [ ] Insomnia/hypersomnia
- [ ] Night terror
- [ ] Sleep disruption
- [ ] Suicide attempt
- [ ] Patient or caregivers have improved the mood
- [ ] Other...

After section 12 Continue to next section

Section 13 of 29

Hyperinsulinemic hypoglycemia

Description (optional)

Do you look after children and young people with hyperinsulinemic hypoglycemia or other forms of *

Yes

No

After section 13 Continue to next section

Section 14 of 29

Hyperinsulinemic hypoglycemia

Description (optional)

What is the estimated number of children and young people aged 0-18 years with hyperinsulinemic *

Short answer text

How do you maintain routine checkup effectively and efficiently during COVID-19 outbreak? *

- As usual, no changes
- Sent SMS and emails for consultations
- Telephone consultations
- Video consultations
- Apps
- Face to face consultation with appropriate personal protective equipment
- Other...

Have you had any patient whose diagnose was delayed due to COVID-19 outbreak? *

- Yes
- No

Have you had the perception that disease management has worsened since COVID-19 outbreak? *

- Yes
- No

Have the percentage of severe hypoglycemia episodes in those cases increased in your practice? *

- Yes
- No

How does your multidisciplinary team deliver education to your patients during the outbreak? *

- [ ] Face to face education wearing appropriate personal protective equipment
- [ ] By telephone
- [ ] Video consultation
- [ ] Via Apps or digital plataforms
- [ ] As usual, no changes
- [ ] Other...

Have any of your patients had shortage of any medical supplies? *

- ◯ Yes
- ◯ No, everything was secured
- ◯ I am not aware of situation

If your patients have had shortage of any medical supply and/or diagnostic tests, please, state here:

Short answer text

Do you have the feeling that parents/families avoid contact with endocrine team because of COVID-19 fear? *

- ◯ Yes
- ◯ No

Do your patients have other comorbidities? *

- ☐ Asthma
- ☐ Cystic fibrosis or broncodisplasia
- ☐ Heart disease
- ☐ Kidney disease
- ☐ Cancer
- ☐ Obesity
- ☐ Hypertension
- ☐ Other...

What was the mean proportion of patients with COVID-19 that exhibited the following? *

	0	1-25%	26-50%	51-75%	76-100%
Fever	○	○	○	○	○
Cough	○	○	○	○	○
Pharyngeal eryt...	○	○	○	○	○
Rhinorrhea	○	○	○	○	○
Shortness of br...	○	○	○	○	○
Headache	○	○	○	○	○
Myalgia	○	○	○	○	○
Gastrointestina...	○	○	○	○	○
Severe Hypogly...	○	○	○	○	○
Asymptomatic	○	○	○	○	○

What was the mean proportion of your patients with COVID-19 who needed the following? *

	0%	1-25%	26-50%	51-75%	76-100%
Admission to h...	○	○	○	○	○
Admission to In...	○	○	○	○	○
Bronchodilator...	○	○	○	○	○
Oxygen	○	○	○	○	○
Noninvasive ve...	○	○	○	○	○

Intubation and ...	○	○	○	○	○
No specific tre...	○	○	○	○	○

What was the mean proportion of your patients with COVID-19 who needed the following specific *

	0%	1-25%	26-50%	51-75%	76-100%
Dose adjustme...	○	○	○	○	○
Antivirals (Rem...	○	○	○	○	○
Anti IL-6	○	○	○	○	○
Hydroxychloroq...	○	○	○	○	○
Azithromicin	○	○	○	○	○

Have you seen psychological problems among children and young people with hyperinsulinemic *

- [ ] None have had psychological problems so far
- [ ] Depression
- [ ] Panic attacks
- [ ] Anxiety
- [ ] Eating disorder
- [ ] Parenting stress
- [ ] Insomnia/hypersomnia
- [ ] Night terror

- [ ] Sleep disruption
- [ ] Suicide attempt
- [ ] Patient or caregivers have improved the mood
- [ ] Other...

After section 14 Continue to next section

Section 15 of 29

Thyroid disorders

Description (optional)

Do you look after children and young people with any thyroid disorders and want to answer to the *

- ( ) Yes
- ( ) No

After section 15 Continue to next section

Section 16 of 29

Thyroid disorders

Description (optional)

What is the estimated number of children and young people aged 0-18 years with any thyroid disorders do *

Short answer text

How do you maintain routine checkup effectively and efficiently during COVID-19 outbreak? *

- [ ] As usual, no changes
- [ ] Sent SMS and emails for consultations
- [ ] Telephone consultations
- [ ] Video consultations
- [ ] Apps
- [ ] Face to face consultation with appropriate personal protective equipment
- [ ] Other...

Have you had any patient whose diagnose was delayed due to COVID-19 outbreak? *

- ( ) Yes
- ( ) No

Have you had the perception that disease management has worsened since COVID-19 outbreak? *

- ( ) Yes
- ( ) No

How does your multidisciplinary team deliver education to your patients during the outbreak? *

- [ ] Face to face education wearing appropriate personal protective equipment

- ☐ By telephone
- ☐ Video consultation
- ☐ Via Apps or digital plataforms
- ☐ As usual, no changes
- ☐ Other...

Have any of your patients had shortage of any medical supplies? *

- ◯ Yes
- ◯ No, everything was secured
- ◯ I am not aware of situation

If your patients have had shortage of any medical supply and/or diagnostic tests, please, state here:

Short answer text

Do you have the feeling that parents/families avoid contact with endocrine team because of COVID-19 fear? *

- ◯ Yes
- ◯ No

Do your patients have other comorbidities? *

- ☐ Asthma
- ☐ [illegible]

- [ ] Cystic fibrosis or broncodisplasia
- [ ] Heart disease
- [ ] Kidney disease
- [ ] Cancer
- [ ] Obesity
- [ ] Hyperthension
- [ ] Other...

What was the mean proportion of patients with COVID-19 that exhibited the following? *

	0	1-25%	26-50%	51-75%	76-100%
Fever	○	○	○	○	○
Cough	○	○	○	○	○
Pharyngeal eryt...	○	○	○	○	○
Rhinorrhea	○	○	○	○	○
Shortness of br...	○	○	○	○	○
Headache	○	○	○	○	○
Myalgia	○	○	○	○	○
Gastrointestina...	○	○	○	○	○
Asymptomatic	○	○	○	○	○

What was the mean proportion of your patients with COVID-19 who needed the following?

What was the mean proportion of your patients with COVID-19 who needed the following: *

	0%	1-25%	26-50%	51-75%	76-100%
Admission to h...					
Admission to In...					
Bronchodilator...					
Oxygen					
Noninvasive ve...					
Intubation and ...					
No specific tre...					

What was the mean proportion of your patients with COVID-19 who needed the following specific *

	0%	1-25%	26-50%	51-75%	76-100%
Dose adjustme...					
Antivirals (Rem...					
Anti IL-6					
Hydroxychloroq...					
Azithromicin					

Have you seen psychological problems among children and young people with thyroid disorders and their *

- [ ] None have had psychological problems so far

- [ ] Depression
- [ ] Panic attacks
- [ ] Anxiety
- [ ] Eating disorder
- [ ] Parenting stress
- [ ] Insomnia/hypersomnia
- [ ] Night terror
- [ ] Sleep disruption
- [ ] Suicide attempt
- [ ] Patient or caregivers have improved the mood
- [ ] Other...

After section 16 Continue to next section

Section 17 of 29

Adrenal disorders

Description (optional)

Do you look after children and young people with any adrenal disorders and want to answer to the *

- ( ) Yes
- ( ) No

After section 17 Continue to next section

Section 18 of 29

Adrenal disorders

Description (optional)

What is the estimated number of children and young people aged 0-18 years with any adrenal disorders do *

Short answer text

How do you maintain routine checkup effectively and efficiently during COVID-19 outbreak? *

- [ ] As usual, no changes
- [ ] Sent SMS and emails for consultations
- [ ] Telephone consultations
- [ ] Video consultations
- [ ] Apps
- [ ] Face to face consultation with appropriate personal protective equipment
- [ ] Other...

Have you had any patient whose diagnose was delayed due to COVID-19 outbreak? *

- ○ Yes
- ○ No

Have you had the perception that disease management has worsened since COVID-19 outbreak? *

- Yes
- No

Have the percentage of adrenal crises episodes in those patients increased in your practice? *

- Yes
- No

How have you been managing these cases in your health care setting? *

- Increase dose and frequency hydrocortisone if were orally taken eg. oral stress dose cover with 20 mg hydrocortisone every 6 h
- Patients on modified release hydrocortisone should switch to immediate release hydrocortisone and take 20 mg orally every 6 h
- If on fludrocortisone, continue at usual dose if stable condition
- Hydrocortisone 100 mg per IV injection in adolescents followed by continuous iv infusion
- Infants and children should receive an initial parenteral injection of 50 mg hydrocortisone/m2 followed by 50 mg/24 h in infants and 100 mg/24 h in children
- Continuous i.v. fluid resuscitation with isotonic saline; regularly check urea and electrolytes
- Other…

How does your multidisciplinary team deliver education to your patients during the outbreak? *

- Face to face education wearing appropriate personal protective equipment
- By telephone

☐ Video consultation

☐ Via Apps or digital plataforms

☐ As usual, no changes

☐ Other...

Have any of your patients had shortage of any medical supplies? *

◯ Yes

◯ No, everything was secured

◯ I am not aware of situation

If your patients have had shortage of any medical supply and/or diagnostic tests, please, state here:

Short answer text

Do you have the feeling that parents/families avoid contact with endocrine team because of COVID-19 fear? *

◯ Yes

◯ No

Do your patients have other comorbidities? *

☐ Asthma

☐ Cystic fibrosis or broncodisplasia

☐ [illegible]

- [ ] Heart disease
- [ ] Kidney disease
- [ ] Cancer
- [ ] Obesity
- [ ] Hyperthension
- [ ] Other…

What was the mean proportion of patients with COVID-19 that exhibited the following? *

	0	1-25%	26-50%	51-75%	76-100%
Fever	○	○	○	○	○
Cough	○	○	○	○	○
Pharyngeal eryt…	○	○	○	○	○
Rhinorrhea	○	○	○	○	○
Shortness of br…	○	○	○	○	○
Headache	○	○	○	○	○
Myalgia	○	○	○	○	○
Gastrointestina…	○	○	○	○	○
Adrenal crisis	○	○	○	○	○
Asymptomatic	○	○	○	○	○

What was the mean proportion of your patients with COVID-19 who needed the following?

[illegible] *

	0%	1-25%	26-50%	51-75%	76-100%
Admission to h...	○	○	○	○	○
Admission to In...	○	○	○	○	○
Bronchodilator...	○	○	○	○	○
Oxygen	○	○	○	○	○
Noninvasive ve...	○	○	○	○	○
Intubation and ...	○	○	○	○	○
No specific tre...	○	○	○	○	○

What was the mean proportion of your patients with COVID-19 who needed the following specific *

	0%	1-25%	26-50%	51-75%	76-100%
Dose adjustme...	○	○	○	○	○
Antivirals (Rem...	○	○	○	○	○
Anti IL-6	○	○	○	○	○
Hydroxychloroq...	○	○	○	○	○
Azithromicin	○	○	○	○	○

Have you seen psychological problems among children and young people with adrenal disorders and their *

- [ ] None have had psychological problems so far

- [ ] Depression
- [ ] Panic attacks
- [ ] Anxiety
- [ ] Eating disorder
- [ ] Parenting stress
- [ ] Insomnia/hypersomnia
- [ ] Night terror
- [ ] Sleep disruption
- [ ] Suicide attempt
- [ ] Patient or caregivers have improved the mood
- [ ] Other...

After section 18 Continue to next section

Section 19 of 29

Calcium-Phosphorus and Bone Metabolism

Description (optional)

Do you look after children and young people with any calcium-phosphorus or bone metabolism disorders *

- ( ) Yes
- ( ) No

After section 19 Continue to next section

Section 20 of 29

Calcium-Phosphorus and Bone Metabolism

Description (optional)

What is the estimated number of children and young people aged 0-18 years with any bone metabolism *

Short answer text

How do you maintain routine checkup effectively and efficiently during COVID-19 outbreak? *

- As usual, no changes
- Sent SMS and emails for consultations
- Telephone consultations
- Video consultations
- Apps
- Face to face consultation with appropriate personal protective equipment
- Other...

Have you had any patient whose diagnose was delayed due to COVID-19 outbreak? *

- Yes
- No

Have you had the perception that disease management has worsened since COVID-19 outbreak? *

- Yes
- No

How does your multidisciplinary team deliver education to your patients during the outbreak? *

- Face to face education wearing appropriate personal protective equipment
- By telephone
- Video consultation
- Via Apps or digital plataforms
- As usual, no changes
- Other...

Have any of your patients had shortage of any medical supplies? *

- Yes
- No, everything was secured
- I am not aware of situation

If your patients have had shortage of any medical supply and/or diagnostic tests, please, state here:

Short answer text

Do you have the feeling that parents/families avoid contact with endocrine team because of COVID-19 fear? *

- ◯ Yes
- ◯ No

Do your patients have other comorbidities? *

- ☐ Asthma
- ☐ Cystic fibrosis or broncodisplasia
- ☐ Heart disease
- ☐ Kidney disease
- ☐ Cancer
- ☐ Obesity
- ☐ Hyperthension
- ☐ Other...

What was the mean proportion of patients with COVID-19 that exhibited the following? *

	0	1-25%	26-50%	51-75%	76-100%
Fever	◯	◯	◯	◯	◯
Cough	◯	◯	◯	◯	◯
Pharyngeal eryt...	◯	◯	◯	◯	◯
Rhinorrhea	◯	◯	◯	◯	◯

Shortness of br…	○	○	○	○	○
Headache	○	○	○	○	○
Myalgia	○	○	○	○	○
Gastrointestina…	○	○	○	○	○
Asymptomatic	○	○	○	○	○

What was the mean proportion of your patients with COVID-19 who needed the following? *

	0%	1-25%	26-50%	51-75%	76-100%
Admission to h…	○	○	○	○	○
Admission to In…	○	○	○	○	○
Bronchodilator…	○	○	○	○	○
Oxygen	○	○	○	○	○
Noninvasive ve…	○	○	○	○	○
Intubation and …	○	○	○	○	○
No specific tre…	○	○	○	○	○

What was the mean proportion of your patients with COVID-19 who needed the following specific *

	0%	1-25%	26-50%	51-75%	76-100%
Dose adjustme…	○	○	○	○	○
Antivirals (Rem…	○	○	○	○	○

Anti IL-6	◯	◯	◯	◯	◯
Hydroxychloroq...	◯	◯	◯	◯	◯
Azithromicin	◯	◯	◯	◯	◯

Have you seen psychological problems among children and young people with bone metabolism disorder *

- ☐ None have had psychological problems so far
- ☐ Depression
- ☐ Panic attacks
- ☐ Anxiety
- ☐ Eating disorder
- ☐ Parenting stress
- ☐ Insomnia/hypersomnia
- ☐ Night terror
- ☐ Sleep disruption
- ☐ Suicide attempt
- ☐ Patient or caregivers have improved the mood
- ☐ Other...

After section 20 Continue to next section

Section 21 of 29

Pituitary gland disorders (including Central

Description (optional)

Do you look after children and young people with any pituitary gland disorders (including CNS-related *

- Yes
- No

After section 21 Continue to next section

Section 22 of 29

Pituitary gland disorders (including Central

Description (optional)

What is the estimated number of children and young people aged 0-18 years with any pituitary gland *

Short answer text

How do you maintain routine checkup effectively and efficiently during COVID-19 outbreak? *

- [ ] As usual, no changes
- [ ] Sent SMS and emails for consultations
- [ ] Telephone consultations
- [ ] Video consultations
- [ ] [illegible]

- [ ] Apps
- [ ] Face to face consultation with appropriate personal protective equipment
- [ ] Other...

Have you had any patient whose diagnose was delayed due to COVID-19 outbreak? *

- ( ) Yes
- ( ) No

Have you had the perception that disease management has worsened since COVID-19 outbreak? *

- ( ) Yes
- ( ) No

How does your multidisciplinary team deliver education to your patients during the outbreak? *

- [ ] Face to face education wearing appropriate personal protective equipment
- [ ] By telephone
- [ ] Video consultation
- [ ] Via Apps or digital plataforms
- [ ] As usual, no changes
- [ ] Other...

Have any of your patients had shortage of any medical supplies? *

- ◯ Yes
- ◯ No, everything was secured
- ◯ I am not aware of situation

If your patients have had shortage of any medical supply and/or diagnostic tests, please, state here:

Short answer text

Do you have the feeling that parents/families avoid contact with endocrine team because of COVID-19 fear? *

- ◯ Yes
- ◯ No

Do your patients have other comorbidities? *

- ☐ Asthma
- ☐ Cystic fibrosis or broncodisplasia
- ☐ Heart disease
- ☐ Kidney disease
- ☐ Cancer
- ☐ Obesity
- ☐ Hyperthension
- ☐ Other...

What was the mean proportion of patients with COVID-19 that exhibited the following? *

	0	1-25%	26-50%	51-75%	76-100%
Fever	○	○	○	○	○
Cough	○	○	○	○	○
Pharyngeal eryt...	○	○	○	○	○
Rhinorrhea	○	○	○	○	○
Shortness of br...	○	○	○	○	○
Headache	○	○	○	○	○
Myalgia	○	○	○	○	○
Gastrointestina...	○	○	○	○	○
Asymptomatic	○	○	○	○	○

What was the mean proportion of your patients with COVID-19 who needed the following? *

	0%	1-25%	26-50%	51-75%	76-100%
Admission to h...	○	○	○	○	○
Admission to In...	○	○	○	○	○
Bronchodilator...	○	○	○	○	○
Oxygen	○	○	○	○	○
Noninvasive ve...	○	○	○	○	○
Intubation and ...	○	○	○	○	○

No specific tre…	◯	◯	◯	◯	◯

What was the mean proportion of your patients with COVID-19 who needed the following specific *

	0%	1-25%	26-50%	51-75%	76-100%
Dose adjustme…	◯	◯	◯	◯	◯
Antivirals (Rem…	◯	◯	◯	◯	◯
Anti IL-6	◯	◯	◯	◯	◯
Hydroxychloroq…	◯	◯	◯	◯	◯
Azithromicin	◯	◯	◯	◯	◯

Have you seen psychological problems among children and young people with pituitary gland disorders and *

- [ ] None have had psychological problems so far
- [ ] Depression
- [ ] Panic attacks
- [ ] Anxiety
- [ ] Eating disorder
- [ ] Parenting stress
- [ ] Insomnia/hypersomnia
- [ ] Night terror
- [ ] Sleep disruption

- [ ] Suicide attempt
- [ ] Patient or caregivers have improved the mood
- [ ] Other...

After section 22 Continue to next section

Section 23 of 29

Growth disorders

Description (optional)

Do you look after children and young people with growth disorders and want to answer to the questions *

- ( ) Yes
- ( ) No

After section 23 Continue to next section

Section 24 of 29

Growth disorders

Description (optional)

What is the estimated number of children and young people aged 0-18 years with growth disorders do you *

Short answer text

How do you maintain routine checkup effectively and efficiently during COVID-19 outbreak? *

- [ ] As usual, no changes
- [ ] Sent SMS and emails for consultations
- [ ] Telephone consultations
- [ ] Video consultations
- [ ] Apps
- [ ] Face to face consultation with appropriate personal protective equipment
- [ ] Other...

Have you had any patient whose diagnose was delayed due to COVID-19 outbreak? *

- ◯ Yes
- ◯ No

Have you had the perception that disease management has worsened since COVID-19 outbreak? *

- ◯ Yes
- ◯ No

How does your multidisciplinary team deliver education to your patients during the outbreak? *

- [ ] Face to face education wearing appropriate personal protective equipment

- [ ] By telephone
- [ ] Video consultation
- [ ] Via Apps or digital plataforms
- [ ] As usual, no changes
- [ ] Other...

Have any of your patients had shortage of any medical supplies? *

- ( ) Yes
- ( ) No, everything was secured
- ( ) I am not aware of situation

If your patients have had shortage of any medical supply and/or diagnostic tests, please, state here:

Short answer text

Do you have the feeling that parents/families avoid contact with endocrine team because of COVID-19 fear? *

- ( ) Yes
- ( ) No

Do your patients have other comorbidities? *

- [ ] Asthma
- [ ] [illegible]

- [ ] Cystic fibrosis or broncodisplasia
- [ ] Heart disease
- [ ] Kidney disease
- [ ] Cancer
- [ ] Obesity
- [ ] Hypertension
- [ ] Other...

What was the mean proportion of patients with COVID-19 that exhibited the following? *

	0	1-25%	26-50%	51-75%	76-100%
Fever	○	○	○	○	○
Cough	○	○	○	○	○
Pharyngeal eryt...	○	○	○	○	○
Rhinorrhea	○	○	○	○	○
Shortness of br...	○	○	○	○	○
Headache	○	○	○	○	○
Myalgia	○	○	○	○	○
Gastrointestina...	○	○	○	○	○
Asymptomatic	○	○	○	○	○

What was the mean proportion of your patients with COVID-19 who needed the following?

What was the mean proportion of your patients with COVID-19 who needed the following: *

	0%	1-25%	26-50%	51-75%	76-100%
Admission to h...					
Admission to In...					
Bronchodilator...					
Oxygen					
Noninvasive ve...					
Intubation and ...					
No specific tre...					

What was the mean proportion of your patients with COVID-19 who needed the following specific *

	0%	1-25%	26-50%	51-75%	76-100%
Dose adjustme...					
Antivirals (Rem...					
Anti IL-6					
Hydroxychloroq...					
Azithromicin					

Have you seen psychological problems among children and young people with growth disorders and their *

- [ ] None have had psychological problems so far

- [ ] Depression
- [ ] Panic attacks
- [ ] Anxiety
- [ ] Eating disorder
- [ ] Parenting stress
- [ ] Insomnia/hypersomnia
- [ ] Night terror
- [ ] Sleep disruption
- [ ] Suicide attempt
- [ ] Patient or caregivers have improved the mood
- [ ] Other...

After section 24 Continue to next section

Section 25 of 29

Puberty disorders

Description (optional)

Do you look after children and young people with puberty disorders and want to answer to the questions *

- Yes
- No

After section 25 Continue to next section

Section 26 of 29

Puberty disorders

Description (optional)

What is the estimated number of children and young people aged 0-18 years with puberty disorders do you *

Short answer text

How do you maintain routine checkup effectively and efficiently during COVID-19 outbreak? *

- [ ] As usual, no changes
- [ ] Sent SMS and emails for consultations
- [ ] Telephone consultations
- [ ] Video consultations
- [ ] Apps
- [ ] Face to face consultation with appropriate personal protective equipment
- [ ] Other...

Have you had any patient whose diagnose was delayed due to COVID-19 outbreak? *

- ( ) Yes
- ( ) No

Have you had the perception that disease management has worsened since COVID-19 outbreak? *

- Yes
- No

How does your multidisciplinary team deliver education to your patients during the outbreak? *
